# Development of Persister-FACSeq: a method to massively parallelize quantification of persister physiology and its heterogeneity

**DOI:** 10.1038/srep25100

**Published:** 2016-05-04

**Authors:** Theresa C. Henry, Mark P. Brynildsen

**Affiliations:** 1 Department of Molecular Biology, Princeton University, Princeton, NJ, 08544, USA; 2 Rutgers Robert Wood Johnson Medical School, Piscataway, NJ, 08854, USA; 3 Department of Chemical and Biological Engineering, Princeton University, Princeton, NJ, 08544, USA

## Abstract

Bacterial persisters are thought to underlie the relapse of chronic infections. Knowledge of persister physiology would illuminate avenues for therapeutic intervention; however, such knowledge has remained elusive because persisters have yet to be segregated from other cell types to sufficient purity. This technical hurdle has stymied progress toward understanding persistence. Here we developed Persister-FACSeq, which is a method that uses fluorescence-activated cell sorting, antibiotic tolerance assays, and next generation sequencing to interrogate persister physiology and its heterogeneity. As a proof-of-concept, we used Persister-FACSeq on a library of reporters to study gene expression distributions in non-growing *Escherichia coli,* and found that persistence to ofloxacin is inversely correlated with the capacity of non-growing cells to synthesize protein. Since Persister-FACSeq can be applied to study persistence to any antibiotic in any environment for any bacteria that can harbor a fluorescent reporter, we anticipate that it will yield unprecedented knowledge of this detrimental phenotype.

Biofilms represent a substantial subset of healthcare-associated infections (HAIs), which cost approximately 100,000 lives and $28-54 billion annually in the United States^1^,^2^. Because biofilms have a tendency to relapse, and the bacteria that re-infect them are often as sensitive to the antibiotic used as the original population, bacterial persisters, rather than resistant mutants, are thought to underlie the ability of biofilms to evade permanent eradication^3^,^4^,^5^. The presence of persisters is identified by biphasic killing kinetics: when the bacterial population is exposed to a supra-lethal dose of antibiotic, the vast majority of cells are rapidly killed, whereas persisters survive and exhibit a slower rate of cell death^5^,^6^. When relieved of antibiotic stress, persisters exit their antibiotic-tolerant state and give rise to a new bacterial population with the same susceptibility to the antibiotic used as the original population^3^. Therapeutically-relevant methods to eliminate persisters could dramatically reduce the propensity of biofilm infections to relapse, and thereby alleviate a large burden from the healthcare system. To illuminate such methods, greater knowledge of persister physiology has proven very useful. For example, knowledge that persisters retain translational activity^7^ led to the discovery of metabolites that can potentiate aminoglycoside killing of *Escherichia coli* and *Staphylococcus aureus* persisters^8^. However, while many studies show that dormancy increases the likelihood a bacterium will be a persister^9^,^10^,^11^,^12^,^13^,^14^, many questions remain regarding the physiology of this phenotype. For example, difficulties associated with segregating persisters from other cell types^15^,^16^ have prevented a *bona fide* transcriptome or metabolome of native persisters to be obtained.

The study of persisters has been hindered by their similarity to more highly-abundant cell types, such as viable-but-non-culturable cells (VBNCs). Both persisters and VBNCs exclude cell death stains (*e.g.*, propidium iodide (PI)), harbor metabolic activity, and remain non-replicating during antibiotic stress^15^,^16^,^17^,^18^,^19^,^20^. However, unlike persisters, VBNCs have lost their culturability and fail to give rise to new bacterial populations^15^,^16^,^17^,^18^,^19^, although revival of some has been noted^21^. Keren and colleagues endeavored to isolate *E. coli* persisters by lysing growing cells with ampicillin followed by sedimentation of bacteria with intact membranes^22^; however, it was later shown that only a fraction of the cells that fail to lyse from β-lactam treatment are persisters^15^,^16^. Keren and colleagues also attempted a similar technique in *Mycobacterium tuberculosis* using D-cycloserine^23^. However, VBNCs were not quantified in the non-lysed sample, and therefore, it remains unknown what proportion of the unlysed cells were persisters. Shah and colleagues used FACS to separate cells with low expression from a ribosomal RNA-fluorescent protein (FP) reporter, reasoning that persisters would fall in the subgroup with low protein synthesis^13^. While this low-fluorescing subpopulation had an approximate 20-fold enrichment in persisters^13^, the majority of the low-fluorescing population still succumbed to ofloxacin (OFL). More recently, Cañas-Duarte and colleagues used a chemo-enzymatic lysis solution to segregate a subpopulation of non-lysing cells; however, what proportion of the segregated cells were persisters (*i.e.*, tolerant to antibiotic) remains unknown^24^.

Without methods to provide high purity samples, high-throughput (HT) techniques (*e.g.*, RNA sequencing) cannot be employed to provide knowledge of naturally-occurring persisters. Given these technical challenges, current methods to interrogate persister physiology rely on microscopy^9^,^10^,^11^ or FACS^12^,^13^,^14^,^25^. While such approaches have provided fundamental knowledge of persister states, they are low-throughput and both time- and labor-intensive, which has inhibited their application to systems-level investigations of the persister phenotype.

Here we sought to develop a HT technique to measure and analyze persister physiology and its heterogeneity. We note that persisters have been found to be highly heterogeneous^3^,^4^,^26^,^27^, and therefore, phenotypic distributions are desirable rather than population averages. To accomplish our goal, we employed a fluorescent reporter library and next generation DNA sequencing (NGS) to massively parallelize the current FACS methodology to measure persister physiology. This method, which we have termed Persister-FACSeq, can be used to study persistence of any bacteria to any antibiotic in any growth condition, provided that the bacteria can harbor a fluorescent reporter. We have performed proof-of-concept experiments with a library of transcriptional reporters and demonstrated the utility of our method by studying naturally-occurring persisters to OFL in stationary phase cultures of *E. coli*. We chose to study stationary phase because it is the phase of growth with the highest abundance of persisters (~1 in 10–100)^28^, it is a non-growing, nutrient-depleted environment, which mirrors the environment inside biofilms^29^, and is a condition in which VBNCs far outnumber persisters (Supplementary Fig. S1) and thus hinder the application of conventional high-throughput analyses (*e.g.*, transcriptomics) for the study of persister physiology. We chose OFL as it is effective against non-growing bacteria^30^ and routinely employed to treat serious infections^31^,^32^. In addition, we focus on natively-formed persisters rather than model systems (*e.g.*, cells engineered to accumulate toxins^33^,^34^,^35^,^36^ or treated with chemicals^37^), to ensure that our study would be more applicable to naturally-occurring persistence. Using Persister-FACSeq, we examined a library of ~100 promoters simultaneously, and found impressive agreement between its results and those with monoculture FACS. By employing hierarchical clustering to analyze gene expression from promoters that exhibited distinct normal cell and persister gene expression patterns, we observed a systems-level trend where persistence to OFL was inversely proportional to promoter activity. Further investigation revealed that non-growing cells that maintain competent transcriptional and translational activity were significantly less likely to give rise to OFL persisters in stationary phase populations. These results demonstrate that Persister-FACSeq can rapidly and accurately assay persister physiology and its heterogeneity, and thereby significantly increase the rate at which knowledge of the persister phenotype can be gained.

## Results

### Current FACS method to study persister gene expression

**Figure 1:**
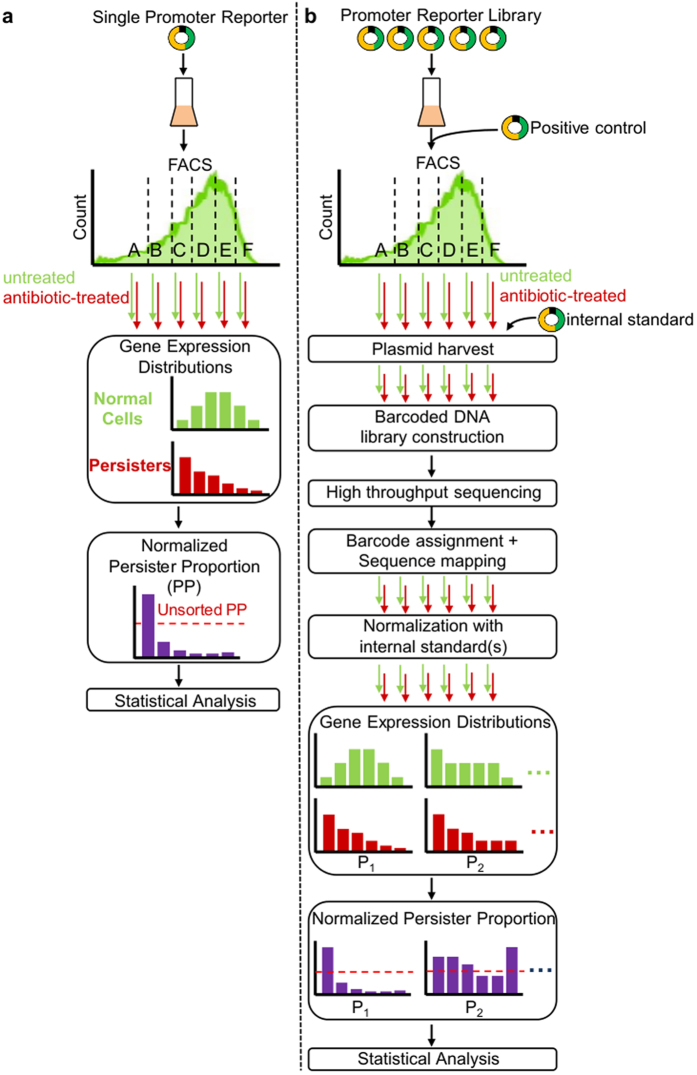
Standard FACS method to measure persister gene expression and parallelized method developed here (Persister-FACSeq). (**a**) Current FACS approach to measure persister gene expression from a single promoter reporter. (**b**) Flow diagram of Persister-FACSeq: a parallelization of approach in (**a**) to measure persister gene expression simultaneously from a library of promoter reporters using FACS and HT sequencing.

Given the lack of biomarkers that can segregate persisters from other cell-types, the current FACS method to study persister gene expression relies on segregation of a population based on promoter activity from fluorescent transcriptional reporters followed by antibiotic tolerance assays to quantify the abundance of persisters across the distribution (Fig. 1a)^12^,^13^,^25^,^38^,^39^. Colony counts from the untreated samples provide the normal (culturable) cell gene expression distribution, whereas those from antibiotic-treated samples provide the persister gene expression distribution. Although this method circumvents the need for persister isolation, it is relatively slow and limited by the range of FPs that can simultaneously be evaluated with FACS. To date, only one to a few promoters have been investigated concurrently^12^,^13^,^25^,^38^.

### NGS-based parallelization of FACS methods to quantify persister gene expression

Here we parallelized the current FACS approach so that gene expression from a library of promoter reporters could be assayed simultaneously (Fig. 1b). With this method, a library is grown together, a positive control is seeded into the population, FACS is performed, and untreated and antibiotic-treated samples are plated. The positive control is a strain harboring a promoter that gives distinct normal cell and persister distributions and is able to be distinguished from other reporter strains when plated (*e.g.*, blue/white colonies). After confirming the anticipated distribution of the positive control (internal control for sorting), colonies are harvested from plates and an internal standard, which is a strain bearing a promoter that was not in the library, is seeded into each sample at equal abundance to facilitate cross quantile comparisons. Plasmids are harvested, promoter regions are amplified with sample-specific barcodes, and the resulting DNA is quantified with NGS. Reads are then segregated by barcode, mapped to promoter sequences, normalized to the internal standard, and promoter abundances in each untreated and treated sample are calculated to yield normal cell and persister gene expression distributions. As an internal control to verify that sequencing results agree with sorting results, the distributions of the positive control are compared. Our method, which we have termed Persister-FACSeq, retains the value of the current FACS approach by circumventing the need for persister isolation, but it is no longer limited by the range of FP colors that can be interrogated simultaneously. Important parameters to consider with this method include persister frequency, fold-change in persistence between quantiles that is desired to be resolvable, and the total number of cells to be sorted. To demonstrate the utility of our method, we studied gene expression of OFL persisters of *E. coli* in stationary phase. Persister frequency dramatically increases as cultures enter stationary phase^28^, but the mechanisms that allow one non-growing, stationary phase cell to become a persister while approximately ninety to ninety-nine of its genetically-identical kin remain sensitive to antibiotic have yet to be determined.

Throughout this work, “persister frequency” is defined as the number of persisters divided by the number of normal (culturable) cells in a population, and is ≤1. We note that persister frequency is equivalent to the survival fraction after sufficient time has passed to reach the second phase of a biphasic kill curve. The “persister proportion” is the proportion of persisters in any subpopulation with respect to all persisters in the population, and is ≤1. The “normalized persister proportion” (*NPP*) is the persister proportion for a subpopulation (specific FACS quantile) divided by the proportion of normal cells that are found within that subpopulation. *NPP*s greater than 1 reflect an over-abundance of persisters within that subpopulation, whereas *NPP*s less than 1 reflect an under-abundance. For a pictorial description of these quantities and their relationship to one another, please see Supplementary Fig. S2.

### Development of a tunable positive control system for use with Persister-FACSeq

**Figure 2:**
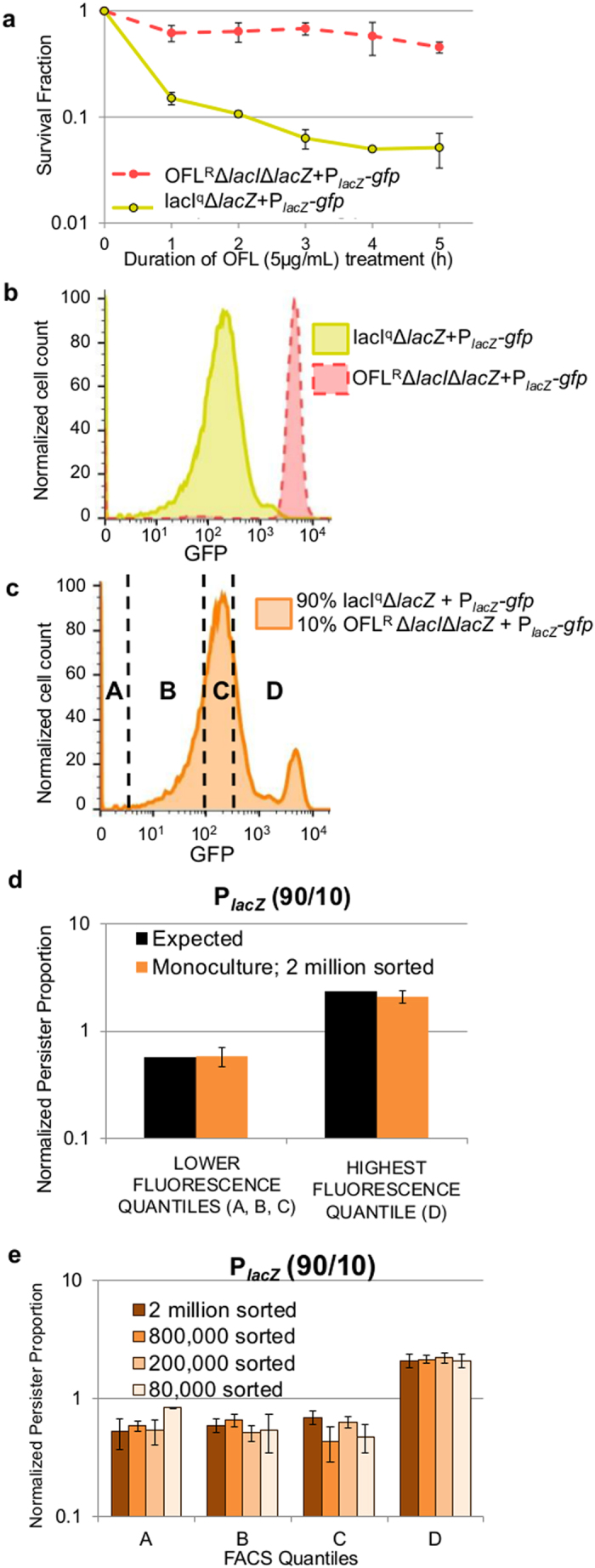
Positive control system. (**a**) Strains OFL^R^Δ*lacI*Δ*lacZ* + P_*lacZ*_*-gfp* and lacI^q^Δ*lacZ* + P_*lacZ*_*-gfp* have survival fractions (persister frequencies) of 0.46 ± 0.05 and 0.05 ± 0.02, respectively, after 5 h treatment with OFL (5 μg/mL) in stationary phase. Data are averages of 3 biological replicates; error bars portray standard error of the mean (s.e.m.). (**b**) Histograms of strains OFL^R^Δ*lacI*Δ*lac*Z + P_*lacZ*_*-gfp* and lacI^q^Δ*lacZ* + P_*lacZ*_*-gfp*. Histograms are representative of 3 biological replicates. **(c)** Histogram of a 90% lacI^q^Δ*lacZ* + P_*lacZ*_*-gfp* and 10% OFL^R^Δ*lacI*Δ*lacZ* + P_*lacZ*_*-gfp* mixture. A-B-C-D designates FACS quantiles used for OFL tolerance assays shown in (**d**,**e**). Quantiles C and D both contain 25% of the population. Quantile A contains either 25% or the smallest possible fraction of the population based on the sorter’s ability to resolve low-fluorescing events, and quantile B contains 25% or the remaining fraction of the population. Histogram is representative of 3 biological replicates. (**d**) *NPP*s of FACS-sorted samples. Expected *NPP*s were calculated based on survival fractions (persister frequencies) of the individual strains after 5 h OFL (5 μg/mL) treatment (**a**) and the knowledge that OFL^R^Δ*lacI*Δ*lacZ* + P_*lacZ*_*-gfp* falls in quantile D. An approximate 4-fold increase in survival in the *HFQ* (quantile D) as compared to the *LFQ*s (quantiles A, B, C) was expected. When 2 million cells were sorted from the total population using FACS, a statistically-significant ~4-fold increase in survival was seen in the *HFQ* as compared to the *LFQ*s (t-test, p-value ≤ 0.05), confirming the expected results. Results are averages of 3 biological replicates; error bars portray s.e.m. (**e**) Positive control system has high *NPP* in *HFQ* (quantile D) as compared to *LFQ*s (quantiles A, B, C). Two million and 200,000 were sorted from a 90/10 lacI^q^/OFL^R^ mixture. 800,000 and 80,000 were sorted from a 90/10 lacI^q^/OFL^R2^ mixture. Data are averages of 3 biological replicates; error bars portray s.e.m.

We were particularly interested in identifying traits that distinguished persisters from non-persisters in non-growing populations. While the conditions we chose were ripe for discovery, because previous studies that found distinct persister and non-persister gene expression distributions were performed in different experimental conditions, it left us without a previously identified promoter we could use as a positive control. Therefore, we sought to design a positive control system for Persister-FACSeq to ensure proper functioning of the assay. Specifically, our design criteria were that it could produce distinct, tunable persister and normal cell distributions and that it could be differentiated from other reporter strains during plating. To accomplish this, we reasoned that a mixture of two strains, which carry the same transcriptional reporter but have markedly different expression from that promoter and markedly different survival when treated with OFL, would satisfy the first criterion, whereas blue/white screening could be used to satisfy the second criterion. To achieve these properties, we chose to use the P_*lacZ*_*-gfp* reporter, perturbations to *lac* genes, and an OFL resistant mutant. For one strain of the positive control system, we deleted both *lacI* and *lacZ* from MG1655, transformed the resultant strain with P_*lacZ*_*-gfp*, and then raised mutants of this strain that were resistant to ≥5 μg/mL OFL by iteratively plating on higher concentrations of OFL (Supplementary Methods). The final strain, OFL^R^Δ*lacI*Δ*lacZ* + P_*lacZ*_*-gfp* (TH002 + P_*lacZ*_*-gfp*) (Supplementary Table S1), grew in the presence of 5 μg/mL OFL under nutritive conditions (Supplementary Fig. S3), tolerated 5 μg/mL OFL well in stationary phase conditionsof (survival of 0.46 ± 0.05) (Fig. 2a), and exhibited high fluorescence (Fig. 2b). For the second strain of the positive control system, we inserted *lacI* under the control of the lacI^q^ promoter in the *attB* site of the Δ*lacI*Δ*lacZ* strain, and then transformed this strain with P_*lacZ*_*-gfp*. The final strain, lacI^q^Δ*lacZ* + P_*lacZ*_*-gfp* (TH004 + P_*lacZ*_*-gfp*) (Supplementary Table S1), exhibited a wild-type level of survival in the presence of OFL (0.05 ± 0.02) (Fig. 2a) and low fluorescence (Fig. 2b). Flow cytometry indicated that, in a mixture consisting of 90% lacI^q^Δ*lacZ* + P_*lacZ*_*-gfp* and 10% OFL^R^Δ*lacI*Δ*lacZ* + P_*lacZ*_*-gfp* (Fig. 2c), the highly-fluorescent OFL^R^ strain would fall in quantile D when sorted. Therefore, the sample from the highest-fluorescence quantile (*HFQ*) (quantile D) would have a higher *NPP*, calculated using the known survival fractions of the two strains (Fig. 2a) (Methods), than lower fluorescence quantiles (*LFQ*s) (quantiles A, B, and C). *NPP*s from the *LFQ* and the *HFQ* are hereafter referred to as *NPP*_*LFQ*_ and *NPP*_*HFQ*_, respectively. We calculated that when FACS and OFL tolerance assays were performed on the 90/10 lacI^q^/OFL^R^ mixture, the expected *NPP*_*LFQ*_ = 0.56, whereas the expected *NPP*_*HFQ*_ = 2.32 (approximately 4-fold higher than *NPP*_*LFQ*_) (Fig. 2d).

A 90/10 lacI^q^/OFL^R^ mixture was sorted, and the quantiles are shown in Fig. 2c. 500,000 (500k) cells were sorted from each quantile, resulting in a total of 2 million cells sorted from the entire population. *NPP*s from all quantiles (A, B, C, D) are shown in Fig. 2e. Since the *LFQ*s have similar *NPP*s, we have represented their data together as one *LFQ* in Fig. 2d (Methods). When 2 million cells were sorted, *NPP*_*LFQ*_ and *NPP*_*HFQ*_ for P_*lacZ*_ had an ~4-fold change, and were significantly different from each other (t-test, p-value ≤ 0.05), confirming the expected results.

With performance of the positive control system demonstrated with one OFL^R^ strain and a total of 2 million cells sorted, we sought to probe the system’s versatility in two ways. First, we sought to determine whether other OFL^R^ strains could be used, and second, we wanted to assess whether a significant 4-fold difference between *NPP*_*LFQ*_ and *NPP*_*HFQ*_ could be resolved with fewer than 2 million cells sorted. To address these questions, we raised a second OFL^R^ strain (OFL^R2^Δ*lacI*Δ*lacZ* + P_*lacZ*_*-gfp*) in a similar manner as the first and assessed these positive control systems with FACS when 800k, 200k, and 80k cells were sorted. We note that OFL^R2^ had a survival fraction (persister frequency) in stationary phase of 0.60 ± 0.04 after 5 h treatment with 5 μg/mL OFL (Supplementary Fig. S4a) and it also falls in the *HFQ* of FACS-sorted samples (Supplementary Fig. S4b,c). When 200k total cells were sorted from the lacI^q^/OFL^R^ mix, *NPP*_*LFQ*_ and *NPP*_*HFQ*_ were significantly different from each other (t-test, p-value ≤ 0.05) and produced the predicted ~4-fold increase in persistence in quantile D (Fig. 2e). With OFL^R2^, the expected *NPP*s for FACS-sorted samples of a 90/10 lacI^q^*/*OFL^R2^ mixture were *NPP*_*LFQ*_ = 0.49 and *NPP*_*HFQ*_ = 2.54 (Supplementary Fig. S4d). When 800k and 80k cells from this 90/10 lacI^q^/OFL^R2^ mixture were sorted, statistically significant ~4-fold differences between *NPP*_*LFQ*_ and *NPP*_*HFQ*_ were recoverable in both cases (t-test, p-value ≤ 0.05) (Fig. 2e and Supplementary Fig. S4d). Given these results, we deemed that a 90/10 mixture of lacI^q^Δ*lacZ* + P_*lacZ*_*-gfp* and OFL^R^Δ*lacI*Δ*lacZ* + P_*lacZ*_*-gfp* was an adequate positive control system to use in conjunction with Persister-FACSeq. Additionally, this data provided information on critical parameters (persister frequency, resolvable fold-change in persister proportion between quantiles, and total number of cells to sort) that could be used to estimate the size of the library to analyze with Persister-FACSeq under the conditions used here. When Persister-FACSeq is used to interrogate persistence in other conditions (*e.g.*, exponential phase), the positive control can likewise inform these decisions of library size and number of cells to sort as a function of the fold-change in persister proportion that is desirable to detect. The persister frequency of the library under the conditions here (stationary phase) was estimated to be approximately 0.011, based upon the survival of MG1655 containing empty pUA66 vector (Supplementary Fig. S1a), and our positive control system data suggested that, for a 4-fold change between FACS quantiles to be distinguishable, sorting 80k cells from each reporter should be sufficient. We note that a 4-fold difference between quantiles may be resolvable with fewer than 80k cells, but 80k was the lowest total population of sorted cells analyzed with the positive control system here and therefore represents a conservative estimate.

### Persister-FACSeq identifies promoters with distinct normal-cell and persister distributions

As a proof-of-concept, we sought to perform Persister-FACSeq on a library of transcriptional reporters. Since, conservatively, 4-fold differences in *NPP*s under the conditions used here were resolvable with 80k cells per reporter per replicate and the sorter used could sort 6 million cells into 6 quantiles in approximately an hour, we elected to assay a library that contained ~150 reporter strains. This required ~12 million cells to be sorted per replicate, and to do this we opted to combine two independent FACS experiments of 6 million sorted cells each into one replicate of Persister-FACSeq (Methods).

Transcriptional reporters of the SOS response, TA systems, and RpoS regulon were obtained from a promoter reporter library (Zaslaver library), which is comprised of promoter-*gfp* fusions on a low-copy plasmid (pSC101 origin of replication)^40^. The *gfp* variant in these plasmids is stable, which should be considered when interpreting results; however, unstable variants and other fluorescent proteins could also be used with Persister-FACSeq. SOS reporters were chosen because persistence to OFL, a DNA-damaging agent^41^, was investigated here, TA systems were included because they are regarded as terminal effectors of persistence^3^, and members of the RpoS regulon were selected because we focused on stationary phase cultures in this work. Further, we limited reporters to those with promoter regions of approximately 600 bp or less in order to ensure compatibility with the Illumina sequencer. In total, 137 reporters were selected to comprise the proof-of-concept library. As a first step, we transformed the library into the wild-type *E. coli* strain used here (Supplementary Table S1). This was done to avoid secondary mutations that may arise in the library from repeated propagation and distribution, as well as to demonstrate that Persister-FACSeq can be performed with strains outside those that comprise such libraries. Zaslaver reporter strains were grown to stationary phase, mixed in equal quantities, and plasmids were harvested and transformed into wild-type *E. coli* MG1655 (Methods). We recognized that this *en masse* plasmid transformation could have resulted in some reporters being lost from the library; however, we note that the downstream NGS quantifies the library’s composition and if any individual promoters are of exceptional importance they can be independently harvested, transformed, and added to libraries.

**Figure 3:**
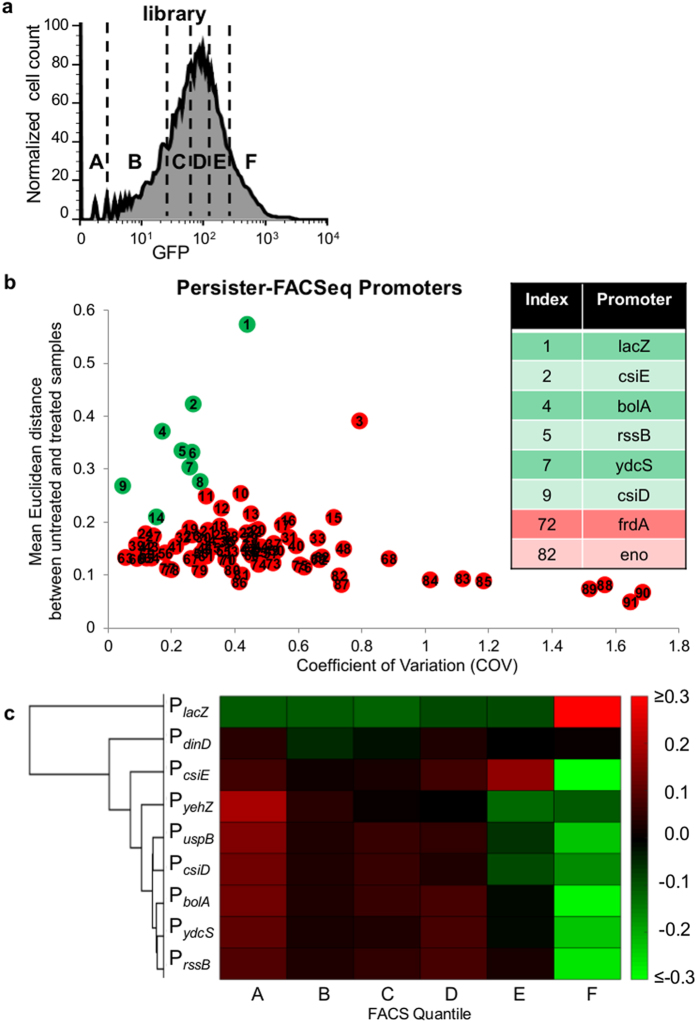
Persister-FACSeq identifies promoters with distinct normal cell and persister gene expression patterns. (**a**) Fluorescence distribution of promoter reporter library [representative of n = 6 (two FACS experiments per Persister-FACSeq replicate, which was performed in triplicate for this study)]. FACS quantiles are designated by A-B-C-D-E-F. Fractions of the entire population sorted into each quantile were in general 0.25, 0.15, 0.15, 0.15, 0.15, 0.15 for A-B-C-D-E-F, respectively. (**b**) Plot of mean Euclidean distance between untreated and treated samples against coefficient of variation (COV) for each promoter (n = 3). Green markers represent promoters with Euclidean distances that are significantly different from Euclidean distances of randomized reads of the same promoter as determined using t-tests and a p-value ≤ 0.05 (Methods) (Supplementary Table S4), which indicates distinct normal cell and persister gene expression patterns. Red markers represent promoters with Euclidean distances not significantly different from Euclidean distances of randomized reads of the same promoter (t-test, p-value > 0.05). Inset lists promoters subjected to monoculture verification. (**c**) Hierarchical clustering of differences between persister proportions and normal cell proportions (***Pprop***and ***Nprop***, colorbar represents the magnitudes of the differences) for promoters identified to have distinct normal cell and persister gene expression patterns. Replicates consist of ≥ 3 independent experiments.

The library was propagated to stationary phase as a single culture, and the positive control system was seeded in at a frequency of 1/138 of the population. FACS, OFL tolerance assays, CFU harvest, inclusion of internal standard, and plasmid harvest were then performed (Methods). The fluorescence distribution of the library and representative gating strategy are shown in Fig. 3a. Promoter regions were amplified with barcode-specific primers (Supplementary Table S2a), and NGS was performed, which yielded reads of 67 or 141 nucleotides (nts) (Methods). Sequences were segregated by barcode and trimmed with Galaxy^42^,^43^,^44^, mapped to promoter sequences with Bowtie2^45^, and counted with Galaxy^42^,^43^,^44^ (Methods) (Supplementary Table S3).

Persister-FACSeq was performed three times on the library. From these results, we found that 95 of the original 137 promoters were transferred using the *en masse* reporter transfer procedure. Five of these reporters were not well represented (≤40 total reads, while all others had >14,000 total reads) and so were excluded from further analyses. Mean Euclidean distances between untreated and treated samples for each promoter were calculated (Methods), and a plot of the mean Euclidean distances against their corresponding coefficient of variation (COV) is shown in Fig. 3b. A high Euclidean distance for a promoter is indicative of a large difference in the distribution between untreated and treated samples, and the COV provides a metric of the proportional variability in the data, where low values are produced by small standard deviations with respect to their means. Promoters that exhibited distinct normal and persister gene expression patterns (green markers in Fig. 3b) were identified as those with significant differences between the Euclidean distances obtained from real data and randomized data (Methods) (t-test, p-value ≤ 0.05), and these notably included the positive control system, P_*lacZ*_. All indices, promoter names, mean Euclidian distances, COVs, and p-values are provided in Supplementary Table S4.

**Figure 4:**
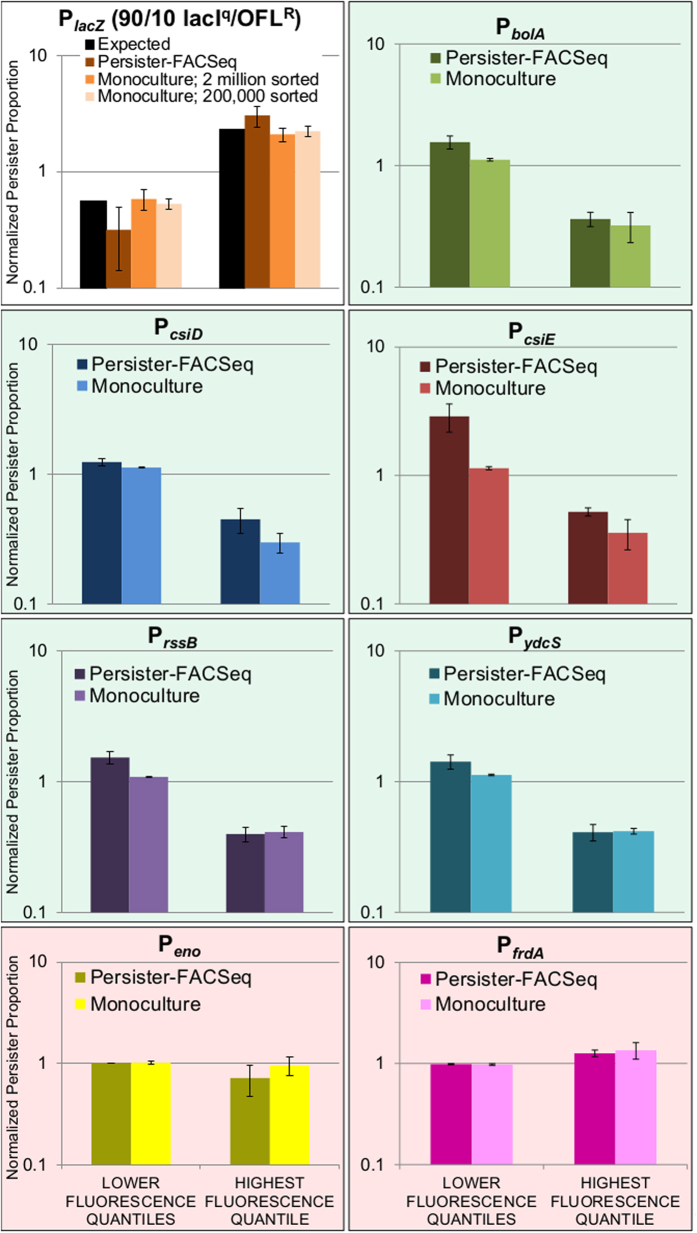
Monoculture *NPP*s confirm Persister-FACSeq *NPP*s for positive control system and all promoters tested. White background indicates positive control system; green background indicates promoters predicted to have distinct normal cell and persister gene expression patterns; red background indicates promoters predicted to not have distinct normal cell and persister gene expression patterns. FACS quantile gating strategies for P_*lacZ*_ and Persister-FACSeq are described in Figs 2 and 3, respectively. FACS quantile gating strategies for monoculture P_*bolA*_, P_*csiD*_, P_*csiE*_, P_*rssB*_, P_*ydcS*_, P_*eno*_, and P_*frdA*_ are described in Supplementary Fig. S5b. Data are averages of 3 biological replicates; error bars portray s.e.m. *NPP*s of each quantile from Persister-FACSeq data are shown in Supplementary Fig. S5a. Controls are shown in Supplementary Fig. S6a.

Interestingly, when hierarchical clustering (Supplementary Methods) was performed on the differences between the vectors that described the distributions of the persister and normal cell proportions, ***Pprop*** and ***Nprop*** (Methods), for promoters with distinct normal cell and persister gene expression patterns, we observed a conspicuous common trend. Namely, other than the positive control, 7 out of the 8 promoters exhibited reduced persistence in the highest fluorescence quantile (Fig. 3c). In order to confirm the accuracy of Persister-FACSeq beyond performance of the positive control system, five promoters with distinct normal cell and persister gene expression patterns that exhibited this trend, P_*bolA*_, P_*csiD*_, P_*csiE*_, P_*rssB*_, and P_*ydcS*_ (green in inset of Fig. 3b), were chosen to be tested with monoculture experiments. These promoters all exhibited 3- to 6-fold lower *NPP*_*HFQ*_s compared to *NPP*_*LFQ*_s (Fig. 4 and Supplementary Fig. S5a), and these differences were significant at a threshold p-value ≤ 0.05 (t-test), except for P_*csiE*_, which had a p-value of 0.08. We also chose two promoters that did not exhibit distinct normal cell and persister gene expression patterns, P_*eno*_ and P_*frdA*_ (red in inset of Fig. 3b), to test in monoculture. These reporters exhibited similar *NPP*s in all quantiles (Fig. 4 and Supplementary Fig. S5a) without any statistically significant differences (t-test, p-value > 0.05).

### Monoculture experiments confirm Persister-FACSeq results

Individual reporter strains were subjected to monoculture FACS and OFL tolerance assays according to the method in Fig. 1a (Methods and Supplementary Methods). All monoculture experiments confirmed trends identified by Persister-FACSeq. P_*bolA*_, P_*csiD*_, P_*csiE*_, P_*rssB*_, and P_*ydcS*_ had low *NPP*_*HFQ*_s, whereas P_*eno*_ and P_*frdA*_ had similar *NPP*s in all quantiles (Fig. 4 and Supplementary Fig. S5b). Differences between *NPP*_*LFQ*_ and *NPP*_*HFQ*_ for P_*bolA*_, P_*csiD*_, P_*csiE*_, P_*rssB*_, and P_*ydcS*_ in monoculture experiments were approximately 3- to 4-fold and significantly different from each other (t-tests, p-value ≤ 0.05), whereas *NPP*_*LFQ*_ and *NPP*_*HFQ*_ for P_*eno*_ and P_*frdA*_ in monoculture experiments were approximately equivalent and not significantly different from each other (t-tests, p-value > 0.05). In addition, we calculated the probability that random selection would perform as well as Persister-FACSeq in properly assigning these 8 promoters to distinct or undistinct gene expression pattern groups to be 0.036 (Supplementary Methods). This demonstrates the utility of Persister-FACSeq to rapidly probe a library of promoter reporters and accurately identify those with distinct normal and persister gene expression patterns.

### Heterogeneous activation of promoters in stationary phase

**Figure 5:**
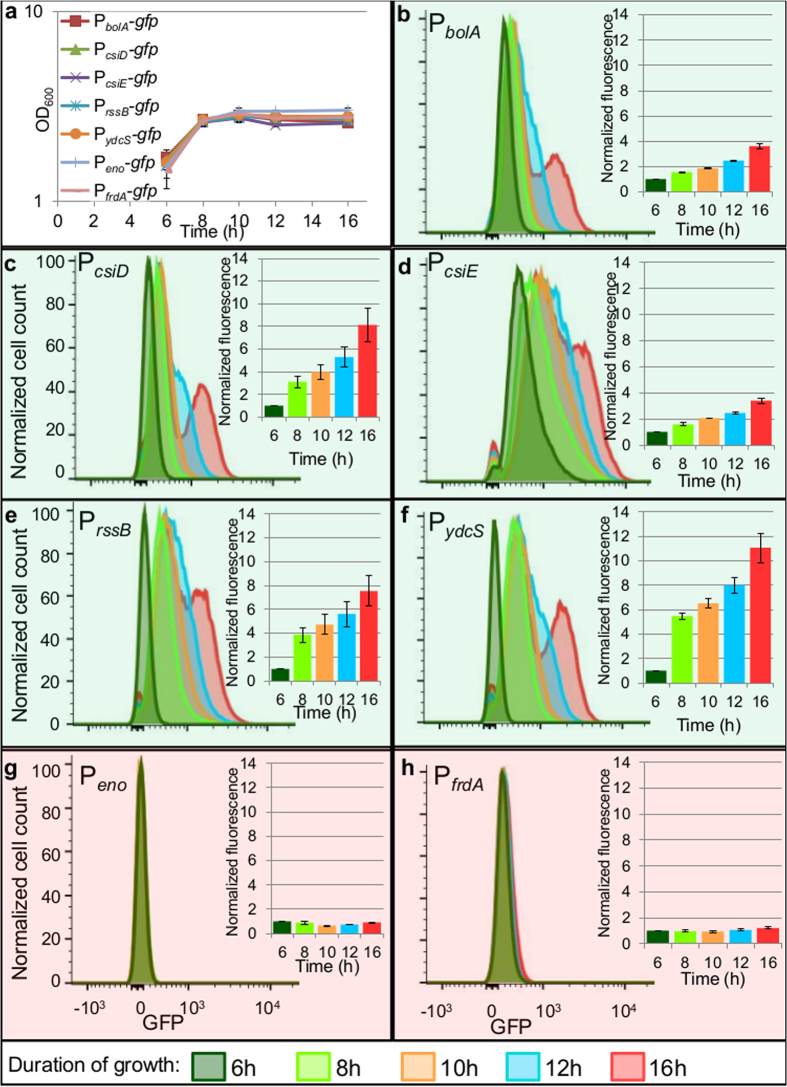
Promoters with distinct normal cell and persister gene expression patterns have a subpopulation that continues to synthesize protein in stationary phase. (**a**) Growth curves of reporter strains show that reporter strains reach stationary phase by ~8 h. Data are averages of 3 biological replicates; error bars portray s.e.m. (**b**–**f**) Promoters with distinct normal cell and persister gene expression patterns contain a subpopulation which continues to actively synthesize protein in stationary phase, as demonstrated by increasing fluorescence between hours 8–16. (**g**,**h**) Promoters not demonstrating distinct normal cell and persister gene expression patterns exhibit constant low fluorescence throughout stationary phase. Histograms are representative of 3 biological replicates. Bar graph insets show fluorescence values normalized to t = 6 h. Graphs represent 3 biological replicates, error bars portray s.e.m.

We observed a common trend for those promoters that exhibited distinct normal and persister gene expression patterns under the conditions tested here (Figs 3c and 4, Supplementary Fig. S5a,b), which was that higher fluorescence produced lower levels of OFL persisters. To investigate this phenomenon further, we first monitored the fluorescence of reporters during growth for 16 h, the time at which FACS and antibiotic tolerance assays had been performed. The cultures reached stationary phase at approximately hour 8 (Fig. 5a), at which time, each population demonstrated a unimodal distribution of fluorescence (light green histograms, Fig. 5), which remained through hour 10 (orange histograms, Fig. 5). By hour 12 (blue histograms, Fig. 5), a subpopulation with higher fluorescence was distinguishable in the promoters with distinct normal and persister gene expression patterns (Fig. 5b–f), and these subpopulations became even more prominent by hour 16. These high-fluorescing subpopulations would have populated the highest fluorescence FACS quantile, and this was confirmed experimentally (Supplementary Fig. S6b). Interestingly, this highly-fluorescent subpopulation was not present in the promoters without distinct normal and persister gene expression patterns (Fig. 5g,h) at any time point we monitored.

### Potential mechanisms underlying the normal cell and persister gene expression patterns observed

Given the expression dynamics we observed for promoters that did and did not exhibit distinct normal cell and persister gene expression patterns (Fig. 5), we reasoned that three potential mechanisms could underlie this phenomenon: 1) higher expression of genes controlled by those promoters reduces persistence to OFL, 2) the results reflect the importance of a shared transcriptional regulator to OFL persistence, and 3) non-growing cells with more competent protein synthesis capabilities, since the fluorescent marker is GFP, are less likely to be persisters to OFL.

### Increased expression of genes controlled by promoters with distinct normal cell and persister patterns does not reduce persistence to OFL

The first hypothesis postulated that persistence to OFL was low in the *HFQ*s of P_*bolA*_, P_*csiD*_, P_*csiE*_, P_*rssB*_, and P_*ydcS*_ because high expression of the genes controlled by those promoters led to low persistence to OFL. To test this hypothesis, we created constructs to overexpress each of the genes that demonstrated distinct normal cell and persister gene expression patterns with the strong, IPTG-inducible T5 promoter on a high-copy plasmid. We confirmed functionality of the constructs by using phenotypic assays, polyacrylamide gel electrophoresis followed by Coomassie Blue staining, mass spectrometry, and/or RT-qPCR (Supplementary Methods and Supplementary Fig. S7). When the genes were over-expressed with 1 mM IPTG at t = 12 h of growth (the time at which subpopulations with continued expression from these promoters became distinguishable in reporters in Fig. 5b–f), persistence to OFL did not significantly decrease as compared to a control overexpressing *mCherry* (t-test, p-value > 0.05) (Supplementary Fig. S8a). These data suggest that a factor other than high expression of specific genes controlled by promoters with distinct normal cell and persister gene expression patterns is responsible for the decreased persistence observed.

### Modulation of RpoS activity does not impact persistence to OFL

The second hypothesis postulated that persistence to OFL was low in the *HFQ*s of P_*bolA*_, P_*csiD*_, P_*csiE*_, P_*rssB*_, and P_*ydcS*_ because those promoters were reporting on the activity of a shared regulator that was important to OFL persistence. Noting that RpoS is the only common regulator of P_*bolA*_, P_*csiD*_, P_*csiE*_, P_*rssB*_, and P_*ydcS*_, we tested the ability of RpoS to affect OFL persister levels by both increasing and decreasing RpoS levels. We decreased RpoS with use of Δ*rpoS*, and increased RpoS levels with both Δ*rssB* (which prevents ClpXP-mediated degradation of RpoS^46^,^47^) and a *rpoS*-overexpression construct (P_*T5*_*-rpoS*) harbored in both wild-type and Δ*rssB* backgrounds. We measured persister frequencies in each strain overexpressing RpoS (induced with 1 mM IPTG as cultures entered stationary phase at OD ~1.5–2) (Supplementary Fig. S8b), and, in all cases, persister frequencies were similar to those of the controls after 5h of OFL treatment (t-test, p-value > 0.05). These data demonstrate that RpoS does not explain the low abundance of persisters in the *HFQ*s of P_*bolA*_, P_*csiD*_, P_*csiE*_, P_*rssB*_, and P_*ydcS*_.

### Active protein production in stationary phase facilitates low tolerance to ofloxacin

**Figure 6:**
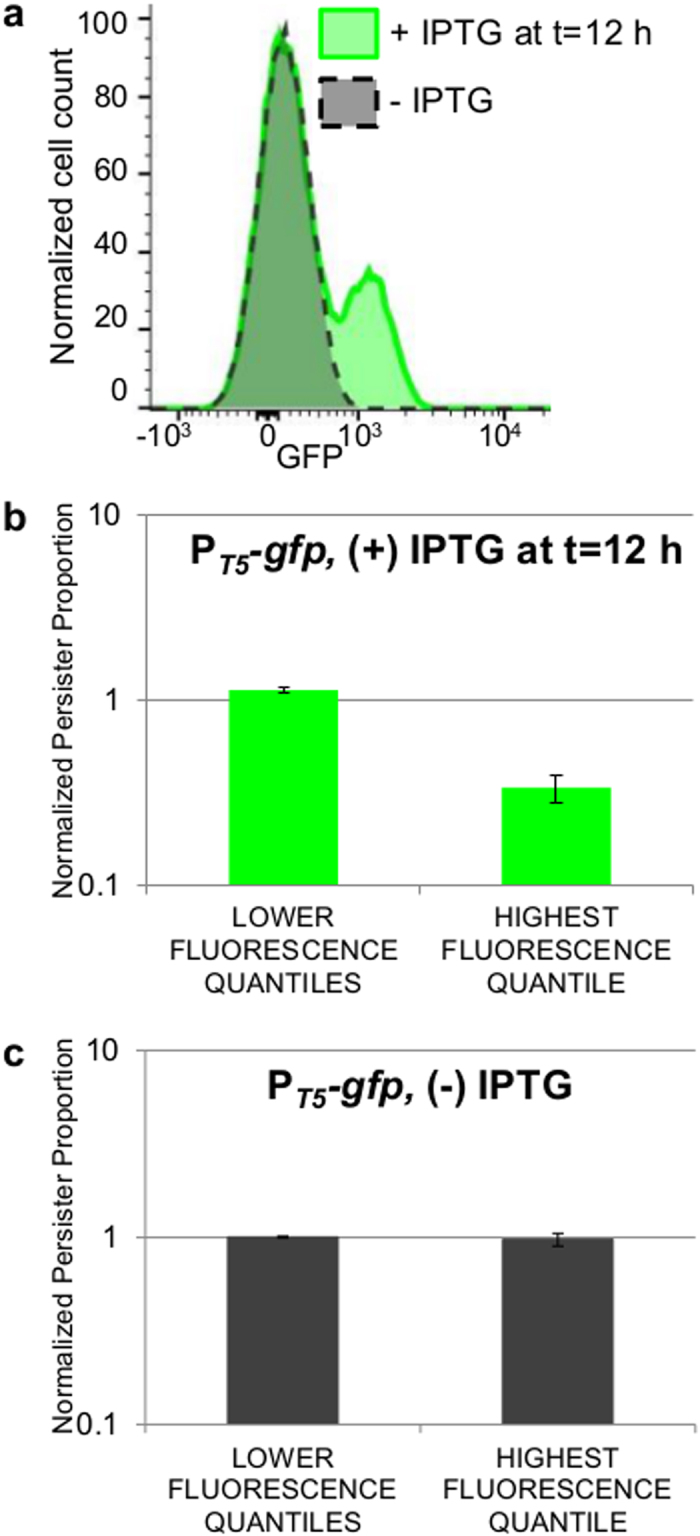
Active protein production in stationary phase correlates with low tolerance to ofloxacin. (**a**) Fluorescence distribution of MG1655 bearing P_*T5*_*-gfp* at t = 16 h (+)/(−) IPTG induction at t = 12 h; representative of 3 biological replicates. (**b**) FACS and OFL tolerance assays were performed on the IPTG-induced P_*T5*_*-gfp* population (green histogram in (**a**)). FACS quantiles were set so that quantile D contained 10% of the entire population, quantile A contained the least number of events possible based on the resolution capabilities of the sorter, quantile C contained <3% of the fluorescent-negative sample (uninduced P_*T5*_*-gfp*), and quantile B contained the remainder of the population. *LFQ*s demonstrate a statistically-significant ~3-fold higher *NPP* than that of the *HFQ* (t-test, p-value ≤ 0.05). Data are averages of 3 biological replicates; error bars portray s.e.m. (**c**) FACS and OFL tolerance assays were performed on an uninduced P_*T5*_*-gfp* population (gray histogram in (**a**)) using the gating strategy described in (**b**) except that quantiles B and C contained equal amounts of the population. *LFQ*s and *HFQ* demonstrate equivalent persistence levels (t-test, p-value > 0.05). Data are averages of 3 biological replicates; error bars portray s.e.m.

The third hypothesis postulated that non-growing cells with more competent protein synthesis capabilities were less likely to exhibit persistence to OFL. To test this hypothesis, we synthetically induced expression of *gfp* in stationary phase using P_*T5*_*-gfp* (pSA21)^48^. Since the subpopulation actively synthesizing protein in the reporters with distinct normal cell and persister gene expression patterns became prominent between hours 12 and 16 of growth (Fig. 5b–f), cultures of MG1655 bearing P_*T5*_*-gfp* were induced with 1 mM IPTG at 12 h of growth and then fluorescence was measured at 16 h. Figure 6a shows that, like the promoters with distinct normal cell and persister gene expression patterns, a subpopulation of the entire culture bearing P_*T5*_*-gfp* actively synthesizes protein when induced at t = 12 h. Further, Fig. 6b shows that the highly-fluorescing subpopulation (*HFQ*) has a lower *NPP* than the rest of the population. The 3.4 fold-change between *NPP*_*LFQ*_ and *NPP*_*HFQ*_ in this synthetically-induced strain is statistically significant (t-test, p-value ≤ 0.05) and, interestingly, very closely mirrors the fold-change in *NPP*s of the promoters with distinct normal cell and persister gene expression patterns, supporting the hypothesis that a general capability for protein synthesis underlies the patterns observed. To ensure that active protein synthesis was responsible for the lower *NPP*_*HFQ*_, we performed analogous experiments on MG1655 + P_*T5*_*-gfp* without IPTG induction (gray histogram, Fig. 6a) and, unlike the induced population, this population did not have a subpopulation actively expressing high protein levels and *NPP*_*LFQ*_ and *NPP*_*HFQ*_ were not significantly different (t-test, p-value > 0.05) (Fig. 6c).

To provide further evidence that the subpopulation primed for general protein expression from the synthetically-inducible promoter was the same subpopulation with high activation of native promoters with distinct normal cell and persister gene expression patterns, we introduced the plasmid containing P_*T5*_*-mCherry*, which fluoresces red when induced with IPTG, into strains already bearing the reporters P_*csiE*_*-gfp* or P_*ydcS*_*-gfp*. Panel 4 of Supplementary Fig. S9b,c shows that the subpopulations with high mCherry levels from the synthetic promoter are the same populations as those with high GFP from the P_*csiE*_ and P_*ydcS*_ promoters. These results demonstrate that, in a population of non-growing bacteria, there is a subpopulation of cells with a relatively higher aptitude for protein synthesis and relatively lower likelihood of giving rise to OFL persisters.

## Discussion

The presence of persisters in bacterial populations is alarming, as they are believed to be the cause of chronic infections. Currently, few methods exist to kill persisters^8^,^49^,^50^,^51^, and none are yet clinically developed. A greater understanding of persister physiology could elucidate novel targets for persister eradication. However, the study of persisters has been hindered by the lack of high-fidelity biomarkers, resulting in an inability to isolate persisters from other more abundant cell types^15^,^16^. Therefore, studies of persister physiology rely on their distinguishing characteristic of survival from lethal antibiotic challenge and growth resumption following antibiotic removal. Unfortunately, this complicates examination of persister physiology, because they are rare and transient and only identified after they exit the persister state. To date, the physiology of natively-formed persisters has largely been interrogated with time-lapse microscopy or FACS^10^,^13^,^25^,^52^, neither of which are well suited to provide systems-level knowledge of or rapidly probe the phenotype. To address this limitation, we developed Persister-FACSeq (Fig. 1b), which quantifies the physiology and heterogeneity of persisters and normal cells simultaneously without the need for persister isolation. Persister-FACSeq only requires that organisms under investigation have the ability to harbor fluorescent reporters (*e.g.*, transcriptional or translational fusions), and the only required infrastructure is a FACS machine and HT sequencer, which are both accessible at most research institutions.

We applied Persister-FACSeq to a proof-of-concept library of transcriptional reporters and confirmed its accuracy with monoculture experiments (Fig. 4). Upon further analysis, we discovered a system-wide trend where, within a non-growing population, there exists a subset of cells with more active protein synthesis, and these bacteria are less likely to be OFL persisters than their other non-growing kin (Figs 5 and 6). This finding of a correlation between active protein synthesis and decreased OFL tolerance in non-replicating bacteria parallels correlations found in exponentially-growing bacteria where high levels of translation, metabolic activity, or replication decreased the likelihood a cell would be a persister^9^,^13^,^14^,^15^. Additionally, our findings are consistent with studies showing that quinolones require that cells have protein and RNA synthesis capabilities for quinolones’ maximum bactericidal capabilities to be achieved^53^,^54^. We postulate that the activity of DNA gyrase, which is the primary target of OFL and is known to participate in RNA synthesis^55^, is different in stationary phase cells with more active protein synthesis when compared to their less active kin, and this renders them more susceptible to killing with OFL.

With the development of Persister-FACSeq, persister physiology and its heterogeneity can be rapidly quantified. The modest requirements of Persister-FACSeq (*e.g.*, organism is fluorescent-reporter compatible, FACS machine, HT sequencer) make it accessible and applicable to a wide variety of studies on bacterial persistence, and further, we demonstrate how a positive control system can be developed and used to delineate critical parameters for application of the method. Since Persister-FACSeq is highly adaptable to the study of other conditions and organisms, and persistence is a phenomenon present in most bacteria tested^56^,^57^,^58^,^59^, we envision that the method will enable unprecedented investigations of persister physiology that will illuminate novel elimination strategies.

## Methods

### Bacterial strains

All strains were derived from *E. coli* K12 MG1655. Strains and plasmids are listed in Supplementary Table S1.

### Antibiotic tolerance assay

Cultures were grown 16 h in LB media at 37 °C with shaking (250 rpm). For overexpression experiments using P_*T5*_*-gfp*, P_*T5*_*-mCherry*, P_*T5*_*-bolA*, P_*T5*_*-csiE*, P_*T5*_*-rssB*, P_*T5*_*-csiD-lhgO-gabDTP*, and P_*T5*_*-ydcSTUV-patD*, cultures were induced with 1 mM IPTG at t = 12 h (the time at which subpopulations with continued expression from promoters with distinct normal cell and persister gene expression patterns became distinguishable from the remainder of the population). For overexpression of RpoS, P_*T5*_*-rpoS* was induced with 1 mM IPTG as cultures reached stationary phase (OD_600_ ~ 1.5–2). After 16 h of growth, all antibiotic tolerance assays were performed under conditions similar to those of FACS-sorted samples. Therefore, all tolerance assays were performed in media that was 50% PBS and 50% sterile-filtered spent LB media (containing 50 μg/mL KAN or 100 μg/mL AMP if needed for plasmid retention), at a cell density of ~200,000 cells/mL. To provide normal (untreated) cell counts, 10 μL of the sample was removed, serially diluted in PBS, and plated. OFL was added to the remaining sample at a final concentration of 5 μg/mL. Samples were then incubated at 37 °C with shaking (250 rpm). Samples were removed at designated time points and centrifuged for 3 min at 15,000 rpm. Supernatant was removed until 100 μL remained and the pellets were resuspended in 900 μL PBS (containing 50 μg/mL KAN if plasmid retention was needed), resulting in an effective 1:10 dilution of OFL concentration. This washing procedure was repeated at least twice more to ensure that the OFL concentration (which was ~0.005 μg/mL after washing) was below the MIC, which was measured to be 0.039–0.078 μg/mL for wild-type MG1655 (Supplementary Methods). Final samples were resuspended in 100 μL PBS (containing 50 μg/mL KAN if plasmid retention was needed), and 10 μL of the sample were serially diluted in PBS and spotted onto LB agar, containing KAN for plasmid retention if needed. Plates were incubated for ~16 h at 37 °C and colony-forming units (CFUs) were counted from the spot containing ~10–50 CFUs. In some cases, additional volume was plated in addition to the 10 μL spots to increase the sensitivity of the assay.

If sorting conditions necessitated that treatments be performed in a total volume ≥3 mL (*i.e.*, total volume in which cells were resuspended post-sorting), OFL was removed from treated samples by first centrifuging the samples at 4,000 rpm for 15 min at room temperature, removing the supernatant leaving ~1 mL sample, resuspending the cells and transferring to a microcentrifuge tube, and then washing according to the procedure described above.

For all FACS-sorted samples, a control was performed to determine the effect of flow through the sorter on persister levels. Cells that were not passed through the sorter were treated under the same conditions as a population that was passed through the cell sorter, and persister frequencies were compared. No significant differences were found between sorted and unsorted samples (Supplementary Fig. S6a).

Unless stated otherwise, two-tailed t-tests with unequal variances were used to assess statistical significance between samples’ persister frequencies.

### Reporter library transformation

Transcriptional reporter plasmid strains from the library generated by Zaslaver and colleagues were used here^40^. The Zaslaver library is a widely accessible resource that can be moved *en masse* at high efficiency (*e.g.*, electroporation), and since it is plasmid based, promoters are flanked by common sequences, which greatly reduce bias in the PCR promoter amplification step that precedes NGS. Further, use of plasmid-based reporters avoids disturbance to the native transcripts (*e.g.*, regulation within the 3′ UTR) and neighboring genes, and numerous persistence studies have used them in conjunction with FACS^12^,^15^,^25^,^38^,^39^; however, we note that chromosomal reporters could also be used with Persister-FACSeq. Reporter strains that report on genes involved in the SOS response, the RpoS regulon, or TA modules, and had promoter regions of approximately 600 bp or less (to facilitate compatibility with Illumina sequencer), were picked and grown individually in wells of a 96-well plate containing 150 μL LB with 50 μg/mL KAN. A total of 135 strains from the Zaslaver library were picked, and two generated in Brynildsen lab, P_*tisB*_^38^ and P_*dinD*_, were added to the collection (Supplementary Table S1). Plates were covered with Breathe-Easy^®^ film and incubated for 16 h at 37 °C with shaking. 10 μL of each strain were combined together and plasmids were harvested using the QIAGEN QIAprep^®^ Spin Miniprep Kit and eluted in 30 μL sterile H_2_O. 1 μL of this plasmid library was transformed into electro-competent wild-type MG1655 and allowed to recover in 1 mL of rich media at 37 °C with shaking for 1 h. 100 μL of this sample was plated on each of 10 LB agar plates containing KAN. Plates were incubated 16–24 h. All CFUs were harvested from all the plates, combined, and stored at −80 °C in 25% glycerol.

### Reporter library FACS, CFU harvest, and plasmid harvest

The library and both strains of the positive control were propagated separately for 16 h. After 16 h, the positive control system (a mixture of 10% OFL^R^Δ*lacI*Δ*lacZ* + P_*lacZ*_*-gfp* and 90% lacI^q^Δ*lacZ* + P_*lacZ*_*-gfp*) was seeded into the library at 1/138 of the entire population. The FACS procedure described in the Supplementary Methods was followed. For replicates 1 and 2, quantiles A-B-C-D-E-F were approximately 25-15-15-15-15-15%, respectively. For replicate 3, the sorter was unable to resolve low-fluorescing events adequately to achieve 25% of the population in quantile A, so quantile A was increased to 30% and quantile B was decreased to 10% to compensate, while quantiles C, D, E, and F contained 15% of the population each. Approximately 1,010,000 cells per quantile were sorted. Sterile-filtered spent media and KAN were added to the sorted samples to result in a final ratio of 50/50 PBS/spent media with 50 μg/mL KAN. Sterile-filtered spent media was used for treatment to render the treatment environment similar to that of stationary phase cultures. The spent media used for treatment consisted of spent LB media from the library with 1/138 of the total volume of a 10/90% mix of spent LB media from the positive control strains added. Approximately 10,000 cells were taken from the sorted sample and diluted into 90 μL PBS with 50 μg/mL KAN (for plasmid retention) and the entire sample was plated. The remaining sample was treated with OFL. After treatment and removal of OFL, the entire sample was plated. Both untreated and treated samples were plated on LB agar containing 50 μg/mL KAN, 1 mM IPTG, and 40 μg/mL Xgal. Corning 245 mm Square BioAssay dishes were used for plating to allow for resolution of single colonies. A total of 12 plates were used for each FACS experiment (one for each treated and untreated sample from each of six quantiles). Additionally, an overnight culture of MG1655 bearing P_*argG*_*-gfp* (a reporter strain not contained in the library) was plated onto a LB agar plate containing KAN at approximately the same CFU density as the library. After 16 h incubation at 37 °C, CFUs were harvested off plates with LB medium containing KAN. An equal volume of MG1655 bearing P_*argG*_*-gfp* was seeded into each of the 12 FACS samples to serve as an internal standard so that quantitative comparison could be made between quantiles. The QIAprep^®^ Spin Miniprep Kit was used to harvest plasmids from 1 mL of this final sample (library including positive control and internal standard) from each quantile. Concentrations of plasmid DNA samples were measured with NanoDrop^TM^ Spectrophotometer ND-1000 and diluted to equal concentrations.

### Sequencing primer design

Primers (Supplementary Table S2a) were designed to be compatible with Illumina HiSeq 2500 sequencer and to amplify promoter regions with sample-specific barcodes. Forward primers consisted of a 58 nt Illumina adapter (italicized in Supplementary Table S2a), a 6 nt barcode unique to each of the 12 samples (bolded in Supplementary Table S2a), and a 20 nt region homologous to a sequence on the pUA66 plasmid 5 base pairs (bps) upstream from the XhoI cut site, which was used for promoter cloning^40^. All samples were amplified with the same reverse primer, which consisted of a 24 nt Illumina adapter (italicized in Supplementary Table S2a) and a 29 nt region homologous to a sequence on the pUA66 plasmid 9 bps downstream from the BamHI cut site, which was used for promoter cloning^40^.

### Preparation of DNA library for sequencing

FACS, antibiotic tolerance assays, and plasmid harvest experiments were performed twice per sequencing experiment. Equal amounts of DNA from the equivalent plasmid minipreps from each of the two separate FACS experiments were combined and 30 ng of each combined sample were used for PCR amplification of promoter regions using primers with sample-specific, sequencing-compatible barcodes. Each PCR tube contained the following: 30 ng combined DNA sample, 2.5 μL of 10 μM forward primer (containing sample-specific barcode and Illumina adapter), 2.5 μL of 10 μM reverse primer (containing Illumina adapter), 1.5 μL DMSO, 1 μL 10 mM dNTPs, 10 μL 5x HF Phusion buffer, 0.5 μL Phusion polymerase, and H_2_O to reach a final volume of 50 μL. The following steps were performed with a thermocycler where the lid temperature was set at 100 °C: 1) 98 °C for 30 secs, 2) 98 °C for 10 secs, 3) 60 °C for 30 secs, 4) 72 °C for 30 secs, 5) GO TO 2, 16 times, 6) 72 °C for 5 mins, 7) Maintain 4 °C. Samples were then purified using the QIAGEN QIAquick PCR purification kit and DNA was eluted with H_2_O. Equal amounts of DNA from all samples were pooled together for sequencing.

### HT sequencing and data analysis

The samples were sequenced as 10% of a single-end 67 nt or 141 nt lane on the Illumina HiSeq 2500 sequencer in Rapid mode following the standard protocol (HiSeq Rapid SBS Kitv2), and raw sequencing reads were filtered by Illumina HiSeq Control Software. Galaxy^42^,^43^,^44^ was used to segregate sequences by barcode (Supplementary Table S2a). For consistency, we only considered the first 67 nts, which included the 6 nt barcode for each sample, the 20 nt sequence from the forward primer homologous to the plasmid backbone, the 5 nt sequence between the primer and the restriction enzyme site, the 6 nt XhoI restriction enzyme site, and the initial 30 nts of the unique promoter constructs used by Zaslaver and colleagues^40^. Galaxy^42^,^43^,^44^ was used to trim sequences to include only nts 48 to 67, and these were mapped to the first 40 bps of the Zaslaver collection^40^ promoter construct sequences using Bowtie2^45^. Promoter sequence abundances were tabulated using Galaxy^42^,^43^,^44^ (Supplementary Table S3).

### Calculation of *NPP*s from monoculture and HT sequencing data

Following FACS and antibiotic tolerance assays, *NPP*s were calculated from CFUs (monoculture experiments) or promoter reads (HT sequencing experiments) in order to provide a comparable metric of persistence between the two methods. For monoculture experiments, CFUs provided persister (*P*) or normal cell (*N*) counts for each promoter *i* in each quantile *j*. For HT sequencing, persister (*P*) and normal cell (*N*) counts for each promoter *i* in each quantile *j* were calculated by normalizing the raw counts for normal cells (*n*) and persisters (*p*) by the raw counts for the internal standard, P_*argG*_, in each quantile using the formulas


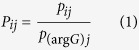


and


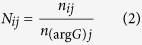


The internal standard was seeded into each sample at equal abundance prior to plasmid extraction in order to account for variations in steps that occur during the independent processing of each sample (*e.g.*, plasmid extraction, preparation of DNA library for sequencing). Persister and normal cell counts were adjusted to take into account the percentage of the entire population within each FACS quantile (*w*_*j*_) with the formulas *w*_*j*_*N*_*ij*_ and *w*_*j*_*P*_*ij*_. Note that 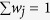. For monoculture experiments, the populations were divided into four FACS quantiles (*e.g.*, Fig. 2c) and *w*_*j*_ are stated within the figures. For the HT method, the population was divided into six FACS quantiles (Fig. 3a), as the distribution was broader than most monoculture distributions. For replicates 1 and 2 of the HT method, *w*_*j*_ was 0.25, 0.15, 0.15, 0.15, 0.15, 0.15 for quantiles A, B, C, D, E, F, respectively. For replicate 3 of the HT method, the percentage of the population that fell in quantile A was increased due to the sorter’s ability to resolve low-fluorescing events. Therefore, for replicate 3, *w*_*j*_ was 0.3, 0.1, 0.15, 0.15, 0.15, 0.15 for quantiles A, B, C, D, E, F, respectively. The persister proportion 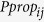 and the normal cell proportion 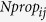 contained in each quantile were calculated using the formulas,


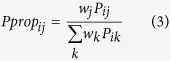


and


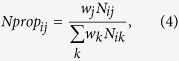


where *k* = {quantiles A, B, C, D} for monoculture experiments and *k* = {quantiles A, B, C, D, E, F} for HT experiments. *NPP*s were calculated using the formula,


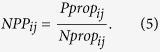


If there was no enrichment or depletion of persisters in a quantile, 
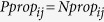
 and *NPP*_*ij*_ = 1. *NPP*s for the combined low fluorescence quantiles (*NPP*_*LFQ*_) were calculated using the formula,


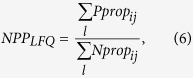


where *l* = {quantiles A, B, C} for monoculture experiments and *l* = {quantiles A, B, C, D, E} for HT experiments. *NPP* for the high fluorescence quantile (*NPP*_*HFQ*_) is


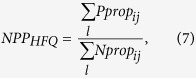


where *l* = {quantile D} for monoculture experiments and *l* = {quantile F} for HT experiments.

### Determination of promoters with distinct normal cell and persister gene expression patterns

In order to determine those promoters, *i*, with the most distinct distributions between normal cells and persisters, the Euclidean distances were calculated using the formula,


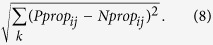


where k are the quantiles defined above.

The mean Euclidean distances for each promoter were then obtained by averaging the Euclidean distances from the three HT sequencing experiments. The coefficient of variation (COV) for each promoter reporter was calculated as the standard deviation of the three Euclidean distances divided by the mean Euclidean distance.

In order to determine promoters with significantly different untreated- and treated-cell distributions, each promoter’s Euclidean distances were compared to Euclidean distances from random distributions of that promoter’s sequences. The random distributions of sequences were generated using Matlab (R2014b). The counts per promoter per quantile was used as an input, and Matlab was used to create two vectors: one containing each promoter represented as an index each time it had been mapped in a quantile, and a second containing the appropriate quantile associated with the promoter in vector 1. A random number was assigned to each promoter index in vector 1 and used to shuffle the promoters in vector 1; promoters were then re-associated to quantiles using the unshuffled second vector. The output was a matrix of promoter reads in each quantile, with the total number of each promoter’s reads conserved but shuffled amongst quantiles. In addition, the total number of reads per quantile was conserved with this procedure. This randomization method was performed on the data from each of the three HT sequencing experiments, and Euclidean distances of each promoter were then determined from the shuffled data of each promoter for each of the three HT sequencing experiments. Significance of the actual data was assessed by comparing the actual Euclidean distances to the randomized Euclidean distances using right-tailed t-tests with unequal variance (p-value ≤ 0.05) and the null hypothesis that Euclidean distances obtained from true sequencing runs would have been equivalent to those from the randomized sequences. Promoters with significantly different Euclidean distances are represented with green markers in Fig. 3b, while all others are represented with red markers.

## Additional Information

**How to cite this article**: Henry, T. C. and Brynildsen, M. P. Development of Persister-FACSeq: a method to massively parallelize quantification of persister physiology and its heterogeneity. *Sci. Rep.* **6**, 25100; doi: 10.1038/srep25100 (2016).

## Supplementary Material

Supplementary Information
