# Supplementary material for: Development of Persister-FACSeq: a method to massively parallelize quantification of persister physiology and its heterogeneity
Source: Sci Rep. 2016 May 4;6:25100. doi: 10.1038/srep25100 (PMC4855238; doi:10.1038/srep25100)
Supplement: Supplementary Information [file srep25100-s1.pdf]

**SUPPLEMENTARY INFORMATION**

**TITLE:** Development of Persister-FACSeq: a method to massively parallelize quantification of persister physiology and its heterogeneity

**AUTHORS:** Theresa C. Henry and Mark P. Brynildsen

**SUPPLEMENTARY FIGURES**

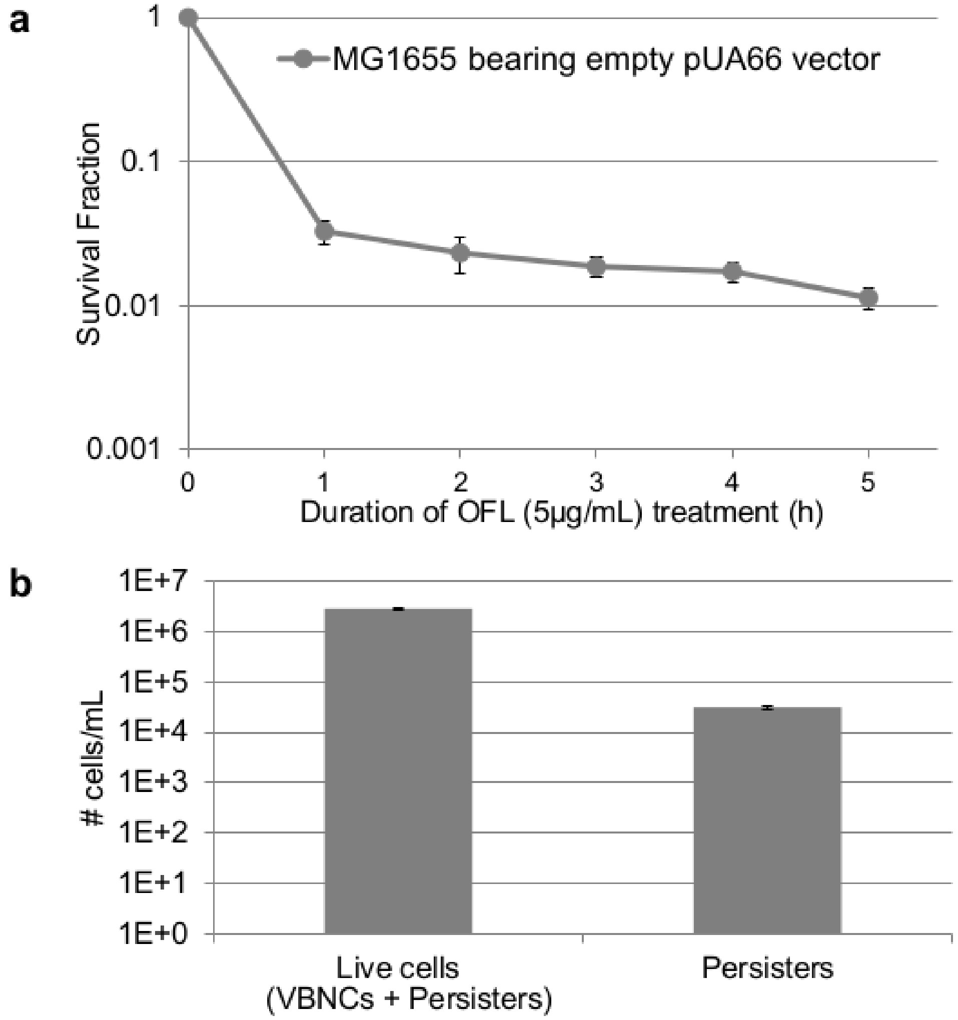


**Supplementary Figure S1. Persister and live cell levels in stationary phase cultures of MG1655 treated with OFL (5μg/mL). (a)** Stationary phase cultures containing empty pUA66 vector have a persister frequency (survival fraction after 5 h OFL treatment) of 0.011±0.0019. Data are averages of 3 independent biological replicates; error bars portray standard error of the mean (s.e.m.). **(b)** Using LIVE/DEAD (SYTO9/PI) staining, counting beads, flow cytometry, and OFL tolerance assays, 2,853,179 ± 61,429 live cells/mL and 31,667 ± 2,186 persisters/mL were identified in OFL-treated stationary phase samples. The concentrations of both persisters and live cells were quantified following antibiotic tolerance assays performed at cell densities and media conditions reflective of samples sorted by fluorescence-activated cell sorting (FACS). Specifically, with the FACS instrument and operating conditions used in this study, sorted samples were provided at a density of ~200,000 cells per 500μL sheath fluid (PBS). These sorted samples were mixed with an equal volume of sterile-filtered, spent media from their respective overnights (for a final concentration of ~200,000 cells/mL), and then treated with 5μg/mL OFL. Therefore, to maintain consistency across experiments, the persister and live cell concentrations reported here are from samples which were treated at an initial density of ~200,000 cells/mL for 5 hours with 5μg/mL OFL in 50% spent media/50% PBS. At the completion of treatment, samples were centrifuged, washed with 0.85% NaCl (as per manufacturer's instructions for SYTO9/PI staining), and concentrated ~10-fold to a final density of ~2,000,000 cells/mL. Concentration of samples minimized background noise (such as electronic noise or small particles in the 0.85% NaCl) during flow cytometry. The number of live cells reflects the cumulative measure of persisters and viable but non-culturable cells (VBNCs). Data are averages of 3 independent biological replicates; error bars portray s.e.m.

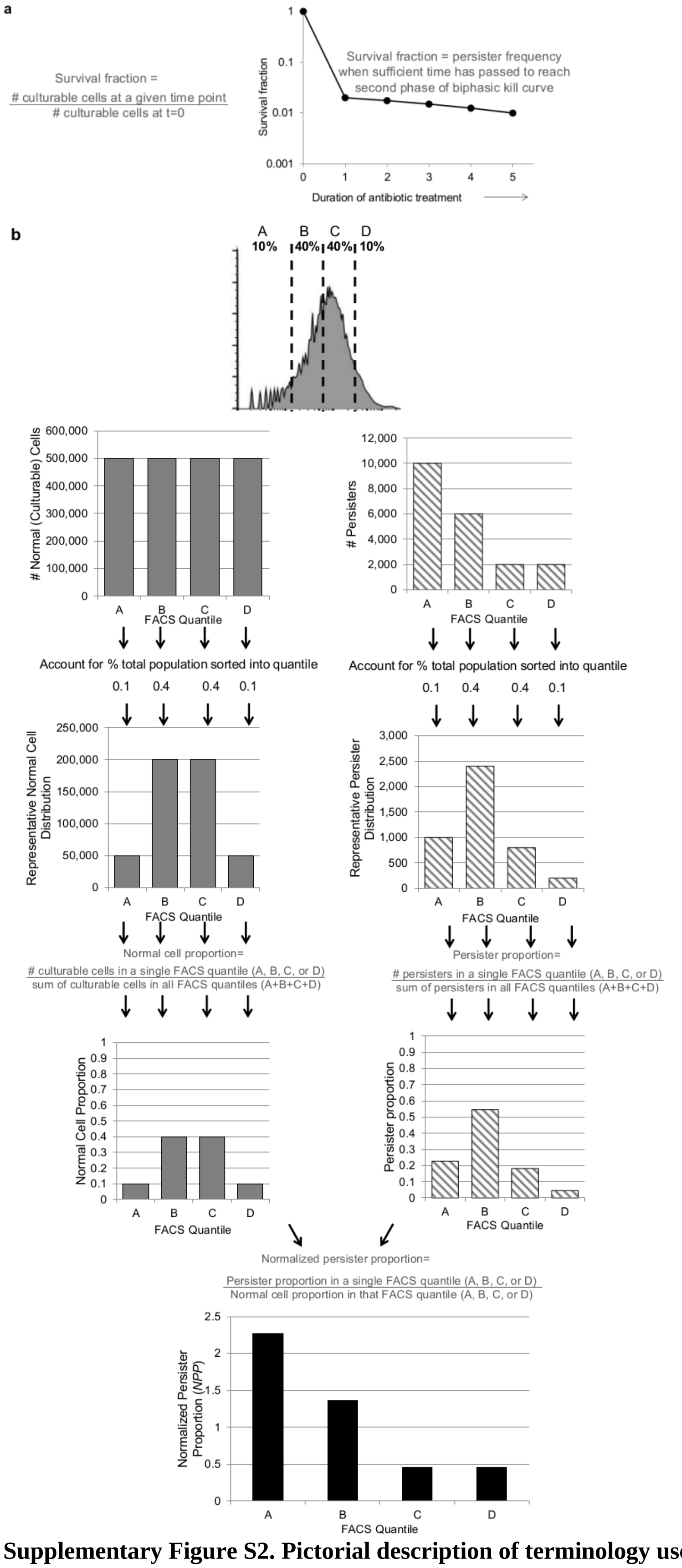


**Supplementary Figure S2. Pictorial description of terminology used in this work.**

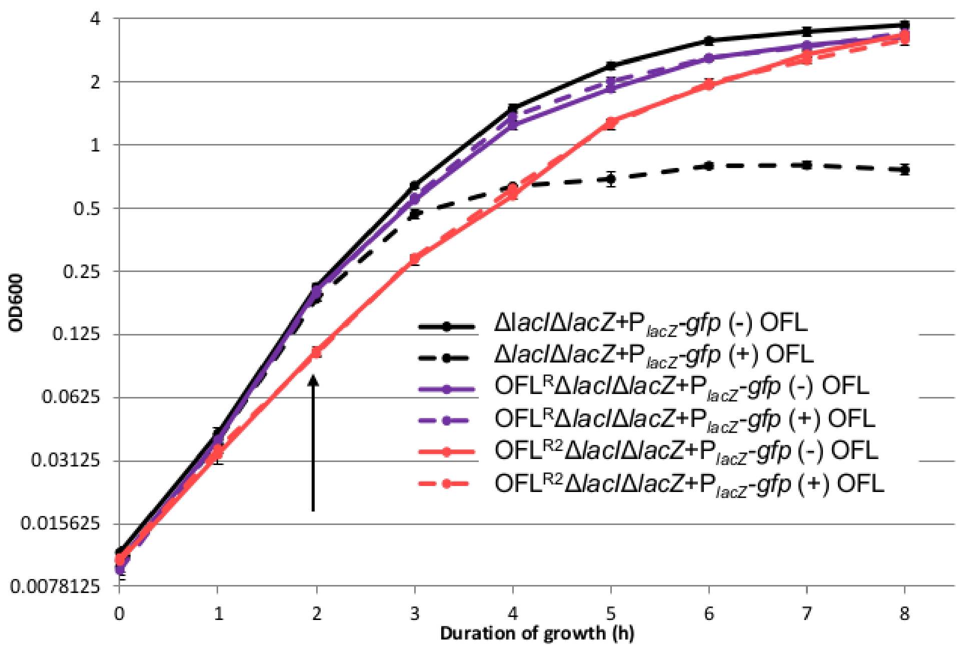


**Supplementary Figure S3. Confirmation of OFL resistance of positive control strains.** $OFL^R$ strains generated for use in the positive control system as well as their parent strain were tested for OFL resistance. 5µg/mL OFL (dashed lines) or the equivalent volume of water (solid lines) was added at t=2h of growth (arrow). $OFL^R$ strains (purple and pink) demonstrate no growth perturbation in the presence of OFL, while their parent strain (black) demonstrates growth inhibition in the presence of OFL.

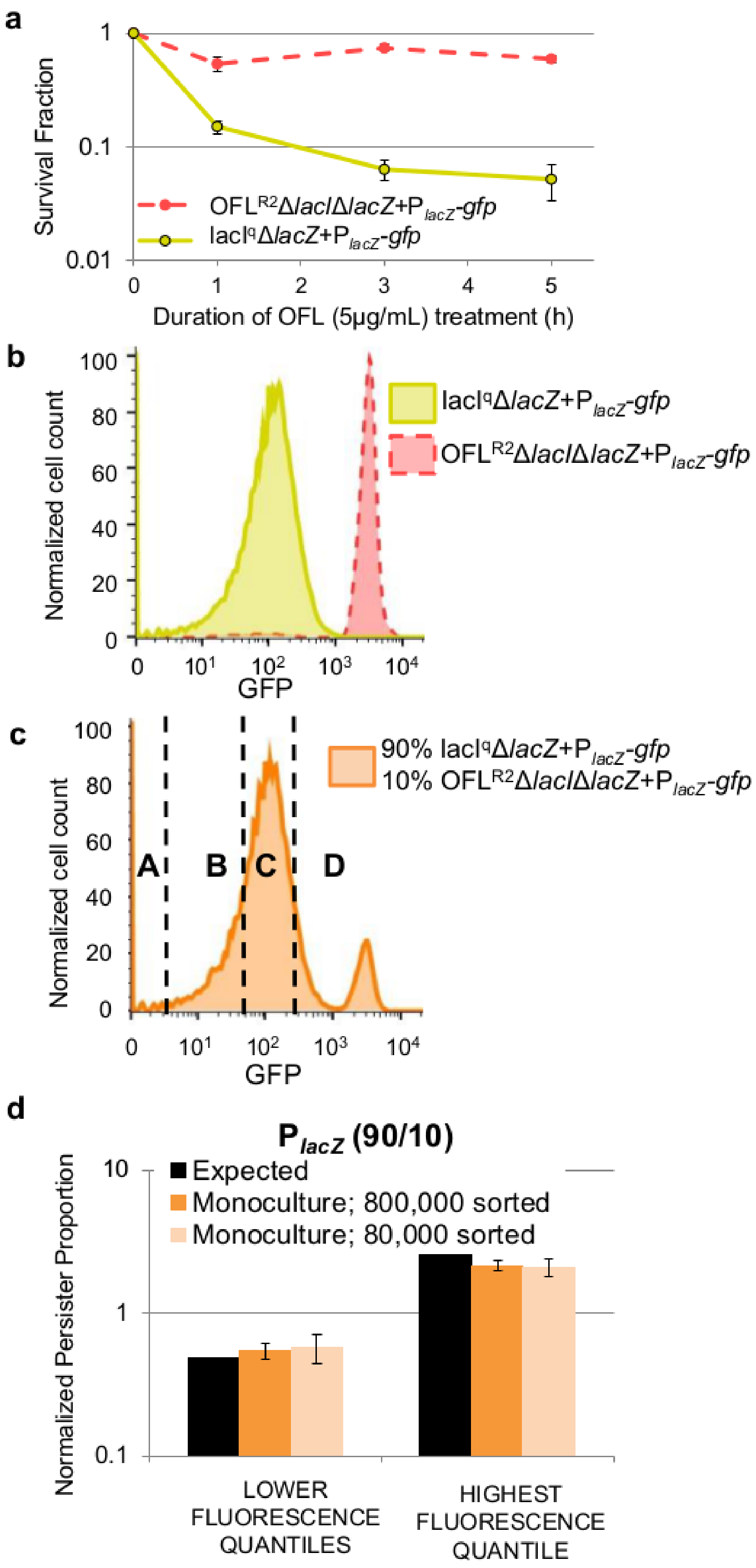
a
1
0.1
0.01
Survival Fraction
OFL^R2ΔlacIΔlacZ+P_lacZ-gfp
lacI^qΔlacZ+P_lacZ-gfp
0 1 2 3 4 5
Duration of OFL (5µg/mL) treatment (h)
b
100 80 60 40 20 0
Normalized cell count
lacI^qΔlacZ+P_lacZ-gfp
OFL^R2ΔlacIΔlacZ+P_lacZ-gfp
0 10^1 10^2 10^3 10^4
GFP
c
100 80 60 40 20 0
Normalized cell count
A B C D
90% lacI^qΔlacZ+P_lacZ-gfp
10% OFL^R2ΔlacIΔlacZ+P_lacZ-gfp
0 10^1 10^2 10^3 10^4
GFP
d
P_lacZ (90/10)
10 1 0.1
Normalized Persister Proportion
Expected
Monoculture; 800,000 sorted
Monoculture; 80,000 sorted
LOWER FLUORESCENCE QUANTILES
HIGHEST FLUORESCENCE QUANTILE

**Supplementary Figure S4. Sorting as few as 80,000 cells from positive control population results in significantly different persister fractions between *LFQ*s and *HFQ*.** Strain $OFL^{R2}$Δ*lacI*Δ*lacZ*+$P_{lacZ}$-*gfp* was raised identically to $OFL^{R}$Δ*lacI*Δ*lacZ*+$P_{lacZ}$-*gfp*. **(a)** Strains $OFL^{R2}$Δ*lacI*Δ*lacZ*+$P_{lacZ}$-*gfp* and $lacI^{q}$Δ*lacZ*+$P_{lacZ}$-*gfp* have persister frequencies (survival fractions after 5 h treatment with 5µg/mL OFL) of 0.60±0.04 and 0.05±0.02, respectively. Results are averages of 3 biological replicates; error bars signify s.e.m. **(b)** Histograms of strains $OFL^{R2}$Δ*lacI*Δ*lac*Z+$P_{lacZ}$-*gfp* and $lacI^{q}$Δ*lacZ*+$P_{lacZ}$-*gfp*. Histograms are representative of 3 biological replicates. **(c)** Histogram of a 90% $lacI^{q}$Δ*lacZ*+$P_{lacZ}$-*gfp* and 10% $OFL^{R2}$Δ*lacI*Δ*lacZ*+$P_{lacZ}$-*gfp* mixture. A-B-C-D designates FACS quantiles used for OFL tolerance assays shown in (d). Quantiles C and D both contain 25% of the population. Quantile A contains either 25% or the smallest possible fraction of the population based on the sorter's ability to resolve low-fluorescing events, and quantile B contains 25% or the remaining fraction of the population. Histogram is representative of 3 biological replicates. **(d)** *NPP*s of FACS-sorted samples. The expected *NPP* was calculated based on persister frequencies of the individual strains (a) and the knowledge that $OFL^{R2}$Δ*lacI*Δ*lacZ*+$P_{lacZ}$-*gfp* would fall in quantile D. When either 800,000 or 80,000 cells were sorted from the total population using FACS, a statistically significant ~4-fold increase in survival was seen in the *HFQ* as compared to the *LFQ*s (t-test, p-value ≤0.05), confirming the expected results. Results are averages of 3 biological replicates; error bars portray s.e.m.

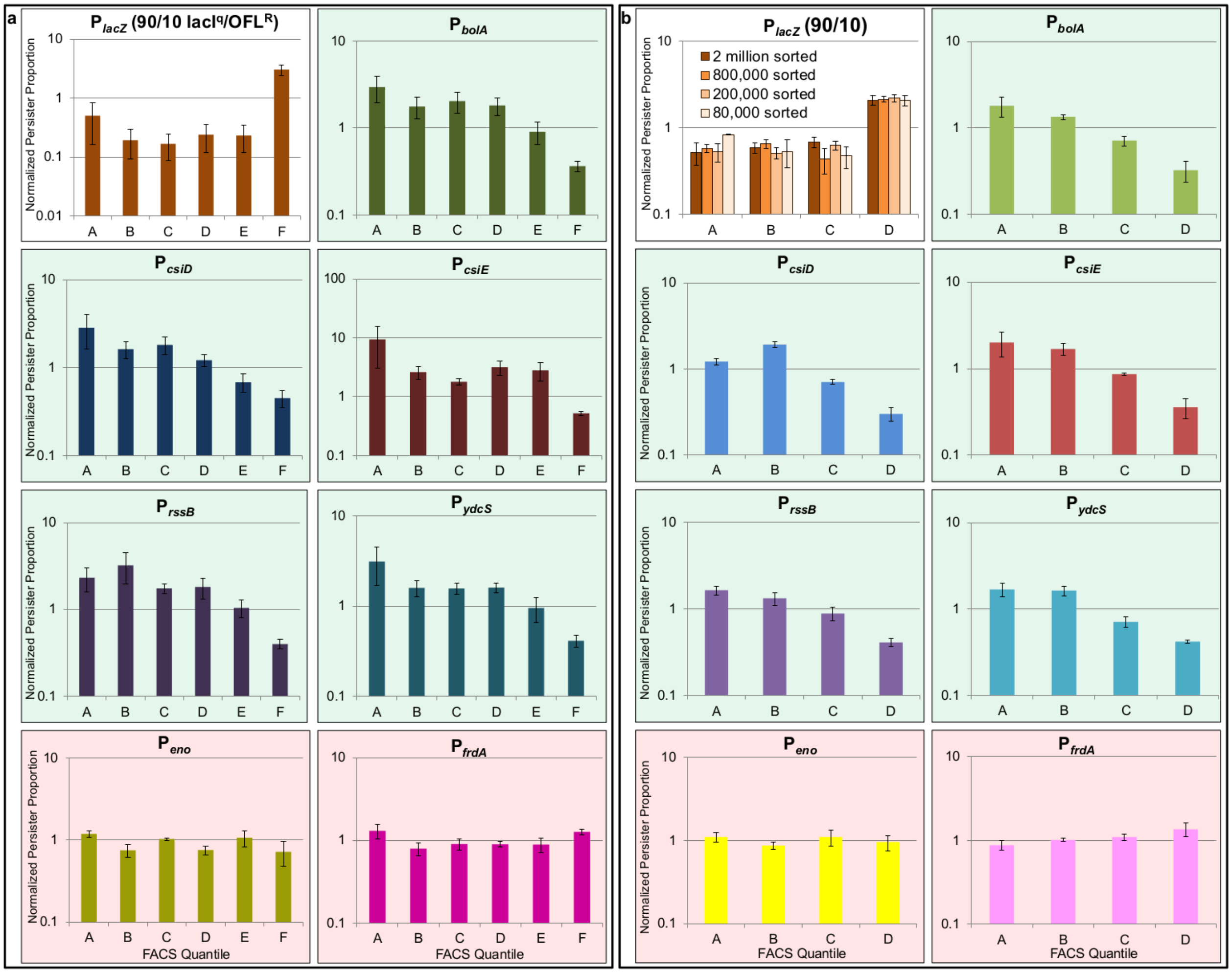


**Supplementary Figure S5**. ***NPPs* from Persister-FACSeq and monoculture experiments. (a) Persister-FACSeq *NPP*s of promoters selected to be tested in monoculture.** Promoters predicted to have distinct normal cell and persister gene expression patterns (green background) demonstrate a common trend of a low *NPP* in the *HFQ* (quantile F) as compared to *NPP*s of *LFQ*s (quantiles A, B, C, D, E). Promoters not predicted to have distinct normal cell and persister gene expression patterns (red background) have similar *NPP*s over all quantiles. Data are averages of 3 biological replicates; error bars portray s.e.m. **(b) *NPP*s from monoculture FACS and OFL tolerance assays.** Promoters predicted by Persister-FACSeq to have distinct normal cell and persister gene expression patterns (green background) demonstrate a common trend of low *NPP* in the *HFQ* (quantile D) as compared to *NPP*s of the *LFQ*s (quantiles A, B, C). Promoters predicted to not have distinct normal cell and persister gene expression patterns (red background) have similar *NPP*s in all quantiles. Positive control is given as a reference. Populations were segregated into A-B-C-D quantiles for monoculture FACS. Quantile D contained 10% of the population; quantile A contained 10% or the lowest percentage resolvable by the cell sorter; quantiles B and C contained equal amounts of the remaining portion of the population. Data are averages of 3 biological replicates; error bars portray s.e.m.

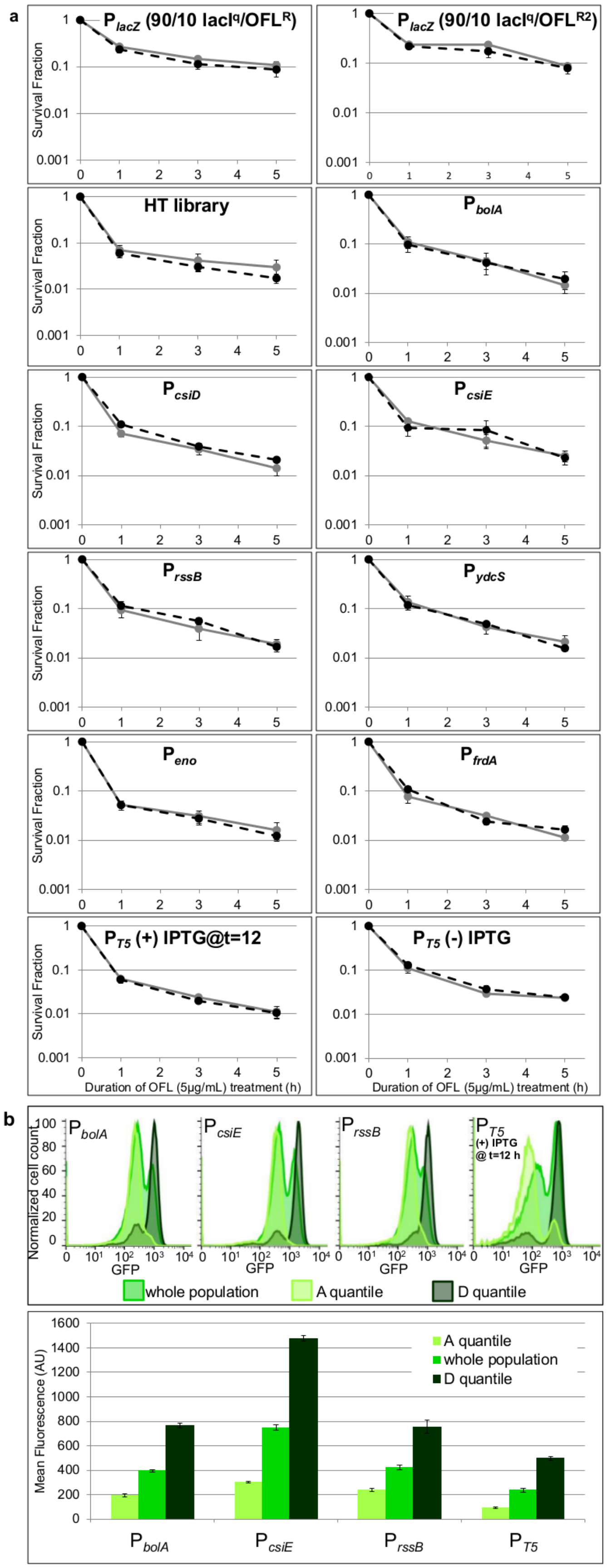
a
P lacZ (90/10 lacI^q/OFL^R)
P lacZ (90/10 lacI^q/OFL^R2)
HT library
P bolA
P csiD
P csiE
P rssB
P ydcS
P eno
P frdA
P T5 (+) IPTG@t=12
P T5 (-) IPTG
Survival Fraction
1
0.1
0.01
0.001
0 1 2 3 4 5
Duration of OFL (5μg/mL) treatment (h)
b
P bolA
P csiE
P rssB
P T5
(+) IPTG @ t=12 h
Normalized cell count
0 20 40 60 80 100
GFP
whole population
A quantile
D quantile
Mean Fluorescence (AU)
0 200 400 600 800 1000 1200 1400 1600
A quantile
whole population
D quantile
P bolA
P csiE
P rssB
P T5

**Supplementary Figure S6. FACS controls. (a) FACS does not change persister frequencies**. To test if sorting affected persister frequency, OFL tolerance assays were performed on samples passed through the cell sorter (solid gray line) and not passed through the cell sorter (dashed black line). There is no significant difference in persister frequencies (survival fractions after 5 h OFL treatment at 5μg/mL) of FACS-sorted and unsorted samples for any reporter (t-test, p-value >0.05). All data represent 3 biological replicates except HT sequencing library (n=6) and $P_{lacZ}$(90/10 lacI$^{q}$/OFL$^{R}$) (n=6). Error bars portray s.e.m. **(b) Reanalysis of FACS-sorted samples.** Samples sorted from quantiles A and D were re-analyzed as described in Supplementary Methods. **Top panel:** Histograms show whole population, FACS-sorted quantile A, and FACS-sorted quantile D samples of promoter reporter strains. Histograms are representative of 3 biological replicates. Almost the entire sample sorted as quantile D is comprised of cells from the high-fluorescing subpopulation of the whole population sample. **Bottom panel:** Mean fluorescence of 3 biological replicates of FACS-sorted promoter reporter strains. AU=arbitrary units. Error bars portray s.e.m. Samples sorted from quantile A have a mean fluorescence lower than that of the entire population, whereas samples sorted from quantile D have a mean fluorescence higher than that of the entire population.

a

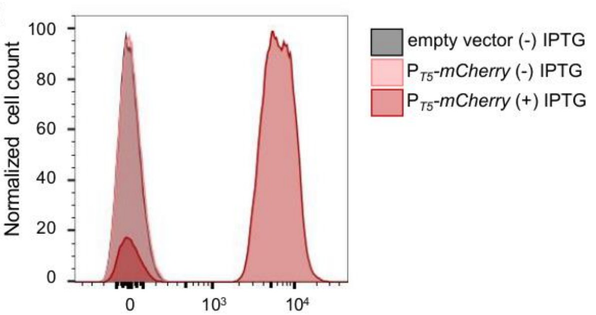


b

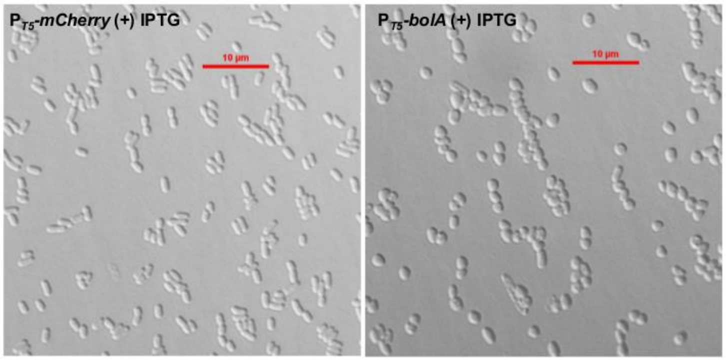


c

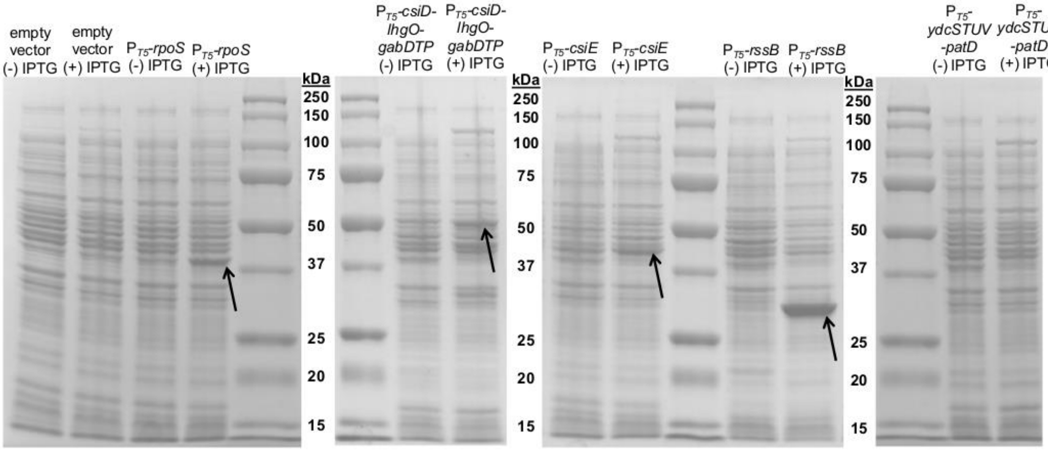


| Identified protein | Total spectrum count in band hypothesized to be CsiE |
|---|---|
| TufB | 449 |
| CsiE | 268 |
| AceA | 251 |
| Eno | 180 |
| Icd | 143 |

| Identified protein | Total spectrum count in band hypothesized to be RssB |
|---|---|
| RssB | 757 |
| Mdh | 49 |
| OmpA | 48 |
| MglB | 46 |
| Tsf | 43 |

d

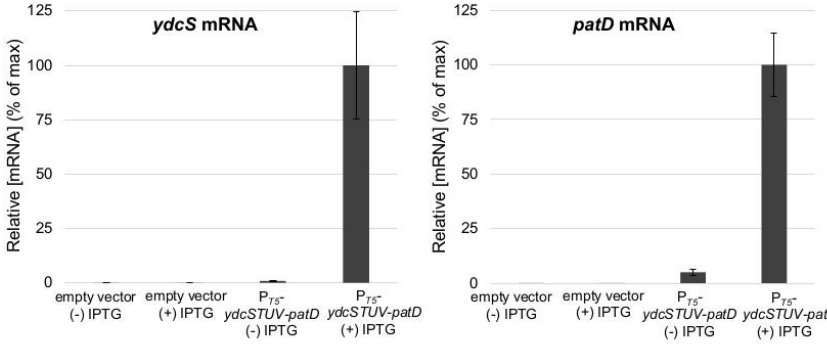

**Supplementary Figure S7. Confirmation of functionality of overexpression constructs.** Phenotypic assays of functionality were used when possible (a, b). In the absence of an available phenotypic assay, functionality was confirmed by observation of increased protein levels (polyacrylamide gel electrophoresis followed by Coomassie Blue staining and, when necessary, mass spectrometry) (c) or transcript levels (RT-qPCR) (d). **(a) Confirmation of functionality of $P_{T5}$-*mCherry*.** Expression of *mCherry* was induced with 1mM IPTG at t=0 of 16 h growth. Histograms of MG1655 bearing $P_{T5}$-*mCherry* (+) or (-) IPTG are shown. Histograms are representative of 3 biological replicates. **(b) Confirmation of functionality of $P_{T5}$-*bolA*.** Microscopy of MG1655 bearing $P_{T5}$-*mCherry* or $P_{T5}$-*bolA* induced with IPTG reveals that overexpression of $P_{T5}$-*bolA* results in the characteristic round-shaped cells[1]. Images are representative of n=2. **(c) Confirmation of functionality of $P_{T5}$-*rpoS*, $P_{T5}$-*csiE*, $P_{T5}$-*rssB*, and $P_{T5}$-*csiD-lhgO-gabDTP*.** Cell lysates of MG1655 bearing $P_{T5}$-*rpoS*, $P_{T5}$-*csiE*, $P_{T5}$-*rssB*, $P_{T5}$-*csiD-lhgO-gabDTP*, and $P_{T5}$-*ydcSTUV-patD* (+) or (-) IPTG were run on a polyacrylamide gel and stained with Coomassie Blue (Supplementary Methods). Hyper-intense bands (arrows) were prominent in the induced samples bearing $P_{T5}$-*rpoS*, $P_{T5}$-*csiE*, $P_{T5}$-*rssB*, and $P_{T5}$-*csiD-lhgO-gabDTP* as compared to the uninduced samples. Image 1: The hyperintense band in MG1655+$P_{T5}$-*rpoS* (+) IPTG ran at ~38kDa, as expected. Image 2: A hyperintense band in MG1655+$P_{T5}$-*csiD-lhgO-gabDTP* (+) IPTG ran at ~50kDa, the approximate molecular weight of two of the proteins (GabD and GabP) in the operon. Image 3: Hyperintense bands were visible in the induced $P_{T5}$-*csiE* and $P_{T5}$-*rssB* samples. Since these bands did not run at the expected molecular weights, the bands were excised and mass spectrometry was performed on the samples (Supplementary Methods). The table below the gels provides the five most abundant *E. coli* proteins identified in each band and their respective abundance (quantified as total spectrum counts). The mass spectrometry data confirms the identity of the proteins in the bands as CsiE and RssB. Image 4: Gel electrophoresis did not reveal hyperintense bands in induced MG1655 bearing $P_{T5}$-*ydcSTUV-patD* as compared to the uninduced control. Therefore, functionality of this construct was confirmed with RT-qPCR. All gel images are representative of ≥2 biological replicates. **(d) Confirmation of functionality of $P_{T5}$-*ydcSTUV-patD*.** RNA from IPTG-induced and uninduced MG1655 bearing $P_{T5}$-*ydcSTUV-patD* or empty vector was isolated, converted to cDNA, and quantified with qPCR (Supplementary Methods). MG1655 bearing $P_{T5}$-*ydcSTUV-patD* (+) IPTG had higher levels of *ydcS* and *patD* RNA as compared to uninduced or empty vector controls. P-values of *ydcS* RNA in $P_{T5}$-*ydcSTUV-patD* (+) IPTG versus $P_{T5}$-*ydcSTUV-patD* (-) IPTG or empty vector (+) IPTG were 0.057 and 0.056, respectively (t-test). P-values of *patD* RNA in $P_{T5}$-*ydcSTUV-patD* (+) IPTG versus $P_{T5}$-*ydcSTUV-patD* (-) IPTG or empty vector (+) IPTG were both 0.02 (t-test). Data are averages of 3 biological replicates; error bars portray s.e.m.

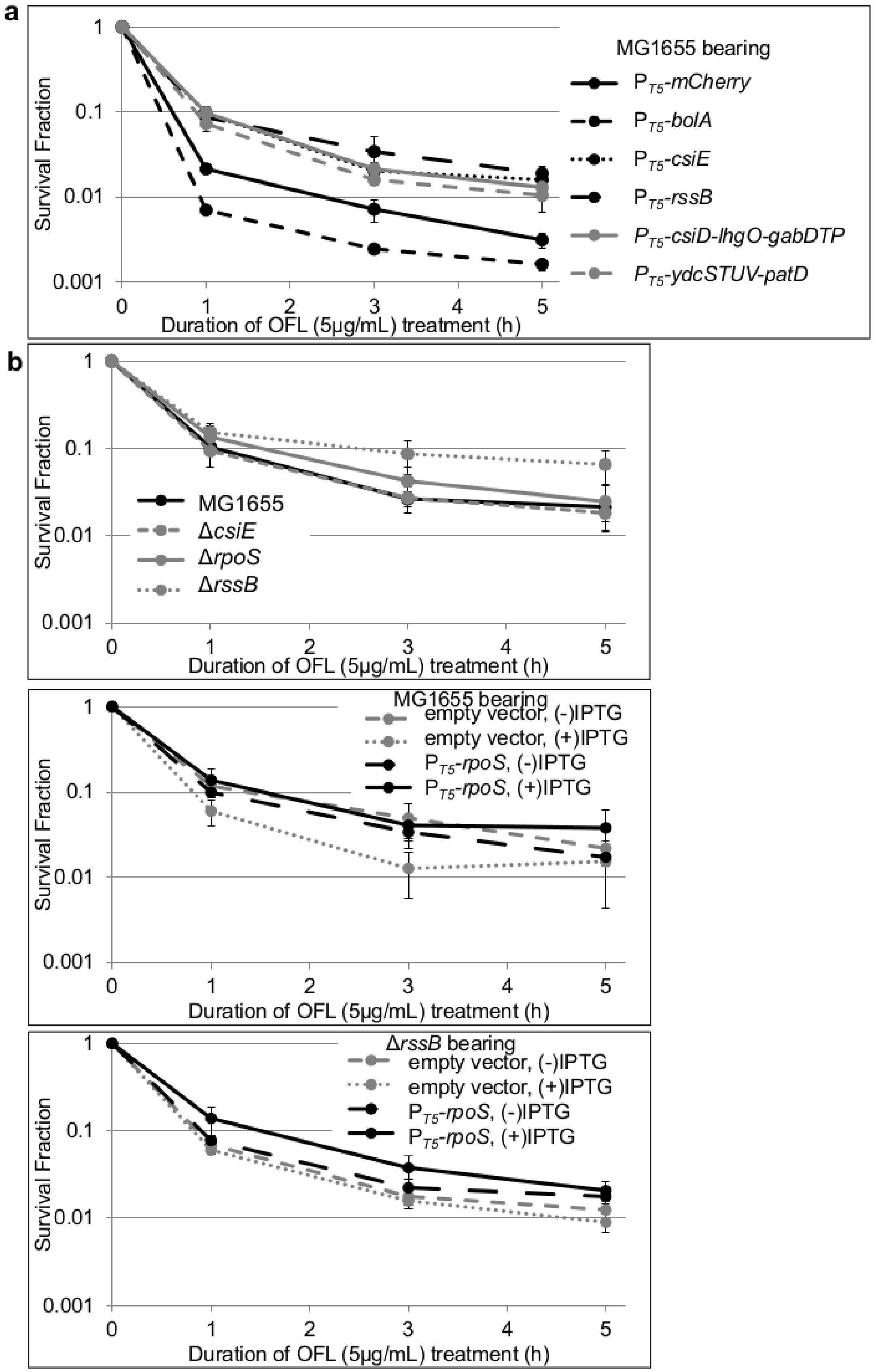
a
b
Survival Fraction
Duration of OFL (5µg/mL) treatment (h)
1
0.1
0.01
0.001
0
1
2
3
4
5
MG1655 bearing
P$_{T5}$-mCherry
P$_{T5}$-bolA
P$_{T5}$-csiE
P$_{T5}$-rssB
P$_{T5}$-csiD-lhgO-gabDTP
P$_{T5}$-ydcSTUV-patD
MG1655
ΔcsiE
ΔrpoS
ΔrssB
empty vector, (-)IPTG
empty vector, (+)IPTG
P$_{T5}$-rpoS, (-)IPTG
P$_{T5}$-rpoS, (+)IPTG
ΔrssB bearing

**Supplementary Figure S8. Antibiotic tolerance assays performed on overexpression and deletion strains. (a)** Genes under control of promoters identified to have distinct normal cell and persister gene expression patterns were overexpressed at t=12 h of growth with 1mM IPTG. Overexpression of *mCherry* is shown for reference. Data are averages of 3 or more biological replicates; error bars signify s.e.m. None of the overexpression strains has a significantly reduced persister frequency when compared to the persister frequency of the *mCherry* overexpression strain (t-test, p-value >0.05). **(b)** We tested if deletion of *csiE* would result in increased persistence, because our FACS data showed that low expression from $P_{csiE}$ resulted in the highest persister levels when compared to low expression from the other promoters with distinct normal cell and persister gene expression patterns (Supplementary Fig. S5). Δ*csiE* did not significantly alter persister levels as compared to wild-type (t-test, p-value >0.05). To test the hypothesis of a common regulator causing the decreased persistence to OFL in the *HFQ*s of $P_{bolA}$, $P_{csiD}$, $P_{csiE}$, $P_{rssB}$, and $P_{ydcS}$, we tested the effect of decreasing and increasing RpoS on OFL persister levels. Deletion of *rpoS* or *rssB* (Panel 1), overexpression of RpoS in wild-type background (Panel 2), and overexpression of RpoS in a Δ*rssB* background (Panel 3) do not alter persister frequencies. RpoS expression from the $P_{T5}$ promoter was induced with 1mM IPTG when cultures reached an optical density of ~1.5-2 (*i.e.,* as cultures entered stationary phase).

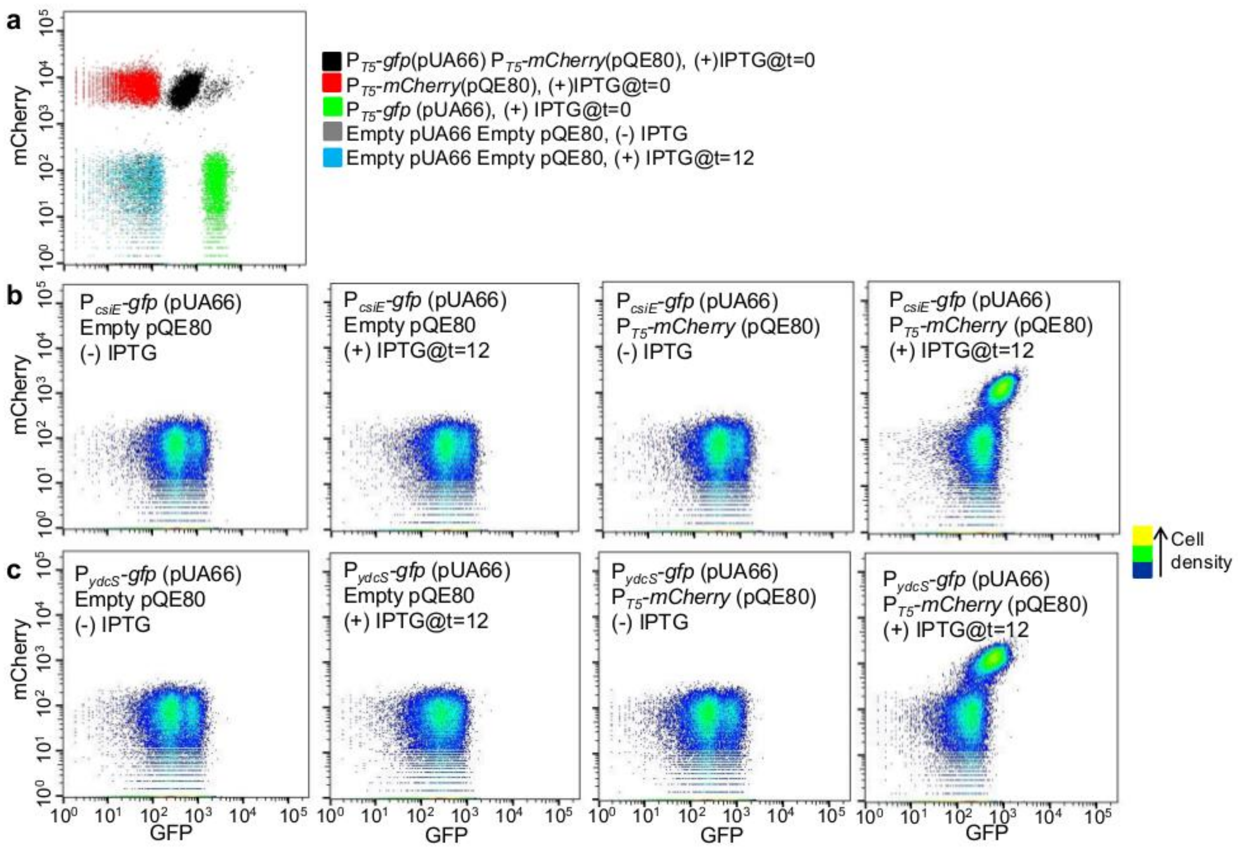


**Supplementary Figure S9. Cells actively synthesizing protein (GFP) from native promoters are primed for protein production when synthetically induced (mCherry). (a)** Red, green, and black dot plots represent fluorescent controls to optimize cytometer settings. Gray and blue dot plots represent empty vector controls (-) or (+) IPTG, respectively, and lack both green and red fluorescence. **(b)** Panels 1 and 2 show MG1655+$P_{csiE}$-*gfp*+empty pQE80 vector (-) or (+) IPTG, respectively. Panel 3 shows MG1655+$P_{csiE}$-*gfp*+$P_{T5}$-*mCherry* (-) IPTG induction. Panel 4 shows MG1655+$P_{csiE}$-*gfp*+$P_{T5}$-*mCherry* (+) 1mM IPTG induction at t=12 h. **(c)** Same as (b) except with $P_{ydcS}$-*gfp* in place of $P_{csiE}$-*gfp*. All cultures were grown for 16 h. For both (b) and (c), panels 1-3 all demonstrate green fluorescence but lack red fluorescence, indicating lack of red autofluorescence or $P_{T5}$-*mCherry* promoter leakiness. Panel 4 of both (b) and (c) shows that the population with high expression from the native promoters (*i.e.*, high GFP) also actively synthesizes protein when induced with IPTG (*i.e.*, high mCherry). Plots are representative of 3 biological replicates.

**SUPPLEMENTARY TABLES**

**Supplementary Table S1. Bacterial strains and plasmids.**

| **Strain** | **Genotype** | **Source or Reference** |
|---|---|---|
| MG1655 | $F^-$ $\lambda^-$ *ilvG*$^-$ *rfb*-50 *rph*-1 | ATCC 700926[2] |
| TH001 | MG1655Δ*lacI*Δ*lacZ* | This work |
| TH002 | MG1655 $OFL^R$Δ*lacI*Δ*lacZ* | This work |
| TH003 | MG1655 $OFL^{R2}$Δ*lacI*Δ*lacZ* | This work |
| TH004 | MG1655Δ*lacI*Δ*lacZ* *attB*::$lacI^q$ $GENT^R$ | This work |
| TH005 | MG1655Δ*rpoS* | P1 Keio[3] → MG1655 |
| TH006 | MG1655Δ*rssB* | This work |
| TH007 | Mg1655Δ*csiE*::$Kan^R$ | P1 Keio[3] → MG1655 |
| **Plasmid** | **Genotype** | **Source or Reference** |
| pUA66 | Vector, SC101 ori, $KAN^R$, *gfpmut2* reporter | Zaslaver and colleagues[4] |
| pQE80 | Vector, ColE1 ori, $P_{T5}$ IPTG-inducible, $AMP^R$ | QIAGEN |
| pKV000 | pUA66+Nhe1, $KAN^R$ | Volzing and Brynildsen[5] |
| pKV010 | pKV000 $P_{tisB}$-*gfpmut2*, $KAN^R$ | Volzing and Brynildsen[5] |
| pTH01 | pKV000 $P_{dinD}$-*gfpmut2*, $KAN^R$ | This work |
| pSA21 | pUA66 *lacI*$^q$ $P_{T5}$-*gfp*, $KAN^R$ | Amato and Brynildsen[6] |
| pTH02 | pQE80 *lacI*$^q$ $P_{T5}$-*mCherry*, $KAN^R$ | Orman and Brynildsen[7] |
| pTH03 | pQE80 *lacI*$^q$ $P_{T5}$-*mCherry*, $AMP^R$ | This work |
| pTH04 | pQE80 *lacI*$^q$ $P_{T5}$-*rpoS*, $AMP^R$ | This work |
| pTH05 | pQE80 *lacI*$^q$ $P_{T5}$-*bolA*, $AMP^R$ | This work |
| pTH06 | pQE80 *lacI*$^q$ $P_{T5}$-*csiE*, $AMP^R$ | This work |
| pTH07 | pQE80 *lacI*$^q$ $P_{T5}$-*rssB*, $AMP^R$ | This work |
| pTH08 | pQE80 *lacI*$^q$ $P_{T5}$-*csiD-lhgO-gabDTP* | This work |
| pTH09 | pQE80 *lacI*$^q$ $P_{T5}$-*ydcSTUV-patD* | This work |
| pUA66 $P_{ada}$ | pUA66 $P_{ada}$-*gfpmut2*, $KAN^R$ | Zaslaver and colleagues[4] |
| pUA66 $P_{adrA}$ | pUA66 $P_{adrA}$-*gfpmut2*, $KAN^R$ | Zaslaver and colleagues[4] |
| pUA66 $P_{aidB}$ | pUA66 $P_{aidB}$-*gfpmut2*, $KAN^R$ | Zaslaver and colleagues[4] |
| pUA66 $P_{aldB}$ | pUA66 $P_{aldB}$-*gfpmut2*, $KAN^R$ | Zaslaver and colleagues[4] |
| pUA66 $P_{aldH}$ | pUA66 $P_{aldH}$-*gfpmut2*, $KAN^R$ | Zaslaver and colleagues[4] |
| pUA66 $P_{argG}$ | pUA66 $P_{argG}$-*gfpmut2*, $KAN^R$ | Zaslaver and colleagues[4] |
| pUA66 $P_{artP}$ | pUA66 $P_{artP}$-*gfpmut2*, $KAN^R$ | Zaslaver and colleagues[4] |
| pUA66 $P_{asr}$ | pUA66 $P_{asr}$-*gfpmut2*, $KAN^R$ | Zaslaver and colleagues[4] |
| pUA66 $P_{astC}$ | pUA66 $P_{astC}$-*gfpmut2*, $KAN^R$ | Zaslaver and colleagues[4] |
| pUA66 $P_{b0257}$ | pUA66 $P_{b0257}$-*gfpmut2*, $KAN^R$ | Zaslaver and colleagues[4] |
| pUA66 $P_{blc}$ | pUA66 $P_{blc}$-*gfpmut2*, $KAN^R$ | Zaslaver and colleagues[4] |
| pUA66 $P_{bolA}$ | pUA66 $P_{bolA}$-*gfpmut2*, $KAN^R$ | Zaslaver and colleagues[4] |
| pUA66 $P_{cbdA}$ | pUA66 $P_{cbdA}$-*gfpmut2*, $KAN^R$ | Zaslaver and colleagues[4] |

| | | |
|---|---|---|
| pUA66 $P_{cbpA}$ | pUA66 $P_{cbpA}$-*gfpmut2*, $KAN^R$ | Zaslaver and colleagues[4] |
| pUA66 $P_{cfa}$ | pUA66 $P_{cfa}$-*gfpmut2*, $KAN^R$ | Zaslaver and colleagues[4] |
| pUA66 $P_{csiD}$ | pUA66 $P_{csiD}$-*gfpmut2*, $KAN^R$ | Zaslaver and colleagues[4] |
| pUA66 $P_{csiE}$ | pUA66 $P_{csiE}$-*gfpmut2*, $KAN^R$ | Zaslaver and colleagues[4] |
| pUA66 $P_{dacB}$ | pUA66 $P_{dacB}$-*gfpmut2*, $KAN^R$ | Zaslaver and colleagues[4] |
| pUA66 $P_{dhaR}$ | pUA66 $P_{dhaR}$-*gfpmut2*, $KAN^R$ | Zaslaver and colleagues[4] |
| pUA66 $P_{dinB}$ | pUA66 $P_{dinB}$-*gfpmut2*, $KAN^R$ | Zaslaver and colleagues[4] |
| pUA66 $P_{dinG}$ | pUA66 $P_{dinG}$-*gfpmut2*, $KAN^R$ | Zaslaver and colleagues[4] |
| pUA66 $P_{dinJ}$ | pUA66 $P_{dinJ}$-*gfpmut2*, $KAN^R$ | Zaslaver and colleagues[4] |
| pUA66 $P_{dkgB}$ | pUA66 $P_{dkgB}$-*gfpmut2*, $KAN^R$ | Zaslaver and colleagues[4] |
| pUA66 $P_{dps}$ | pUA66 $P_{dps}$-*gfpmut2*, $KAN^R$ | Zaslaver and colleagues[4] |
| pUA66 $P_{ecpD}$ | pUA66 $P_{ecpD}$-*gfpmut2*, $KAN^R$ | Zaslaver and colleagues[4] |
| pUA66 $P_{ego}$ | pUA66 $P_{ego}$-*gfpmut2*, $KAN^R$ | Zaslaver and colleagues[4] |
| pUA66 $P_{eno}$ | pUA66 $P_{eno}$-*gfpmut2*, $KAN^R$ | Zaslaver and colleagues[4] |
| pUA66 $P_{evgA}$ | pUA66 $P_{evgA}$-*gfpmut2*, $KAN^R$ | Zaslaver and colleagues[4] |
| pUA66 $P_{fadL}$ | pUA66 $P_{fadL}$-*gfpmut2*, $KAN^R$ | Zaslaver and colleagues[4] |
| pUA66 $P_{fliY}$ | pUA66 $P_{fliY}$-*gfpmut2*, $KAN^R$ | Zaslaver and colleagues[4] |
| pUA66 $P_{frdA}$ | pUA66 $P_{frdA}$-*gfpmut2*, $KAN^R$ | Zaslaver and colleagues[4] |
| pUA66 $P_{ftsK}$ | pUA66 $P_{ftsK}$ -*gfpmut2*, $KAN^R$ | Zaslaver and colleagues[4] |
| pUA66 $P_{ftsQ}$ | pUA66 $P_{ftsQ}$-*gfpmut2*, $KAN^R$ | Zaslaver and colleagues[4] |
| pUA66 $P_{gadB}$ | pUA66 $P_{gadB}$-*gfpmut2*, $KAN^R$ | Zaslaver and colleagues[4] |
| pUA66 $P_{gadX}$ | pUA66 $P_{gadX}$-*gfpmut2*, $KAN^R$ | Zaslaver and colleagues[4] |
| pUA66 $P_{galE}$ | pUA66 $P_{galE}$-*gfpmut2*, $KAN^R$ | Zaslaver and colleagues[4] |
| pUA66 $P_{glgS}$ | pUA66 $P_{glgS}$-*gfpmut2*, $KAN^R$ | Zaslaver and colleagues[4] |
| pUA66 $P_{glpX}$ | pUA66 $P_{glpX}$-*gfpmut2*, $KAN^R$ | Zaslaver and colleagues[4] |
| pUA66 $P_{gmr}$ | pUA66 $P_{gmr}$-*gfpmut2*, $KAN^R$ | Zaslaver and colleagues[4] |
| pUA66 $P_{gyrB}$ | pUA66 $P_{gyrB}$-*gfpmut2*, $KAN^R$ | Zaslaver and colleagues[4] |
| pUA66 $P_{hdeA}$ | pUA66 $P_{hdeA}$-*gfpmut2*, $KAN^R$ | Zaslaver and colleagues[4] |
| pUA66 $P_{hdfR}$ | pUA66 $P_{hdfR}$-*gfpmut2*, $KAN^R$ | Zaslaver and colleagues[4] |
| pUA66 $P_{hipB}$ | pUA66 $P_{hipB}$-*gfpmut2*, $KAN^R$ | Zaslaver and colleagues[4] |
| pUA66 $P_{hofM}$ | pUA66 $P_{hofM}$-*gfpmut2*, $KAN^R$ | Zaslaver and colleagues[4] |
| pUA66 $P_{hrpB}$ | pUA66 $P_{hrpB}$-*gfpmut2*, $KAN^R$ | Zaslaver and colleagues[4] |
| pUA66 $P_{ihfB}$ | pUA66 $P_{ihfB}$-*gfpmut2*, $KAN^R$ | Zaslaver and colleagues[4] |
| pUA66 $P_{ilvY}$ | pUA66 $P_{ilvY}$-*gfpmut2*, $KAN^R$ | Zaslaver and colleagues[4] |
| pUA66 $P_{insC-5}$ | pUA66 $P_{insC-5}$-*gfpmut2*, $KAN^R$ | Zaslaver and colleagues[4] |
| pUA66 $P_{insN-1}$ | pUA66 $P_{insN-1}$-*gfpmut2*, $KAN^R$ | Zaslaver and colleagues[4] |
| pUA66 $P_{katE}$ | pUA66 $P_{katE}$-*gfpmut2*, $KAN^R$ | Zaslaver and colleagues[4] |
| pUA66 $P_{lacZ}$ | pUA66 $P_{lacZ}$-*gfpmut2*, $KAN^R$ | Zaslaver and colleagues[4] |
| pUA66 $P_{lexA}$ | pUA66 $P_{lexA}$-*gfpmut2*, $KAN^R$ | Zaslaver and colleagues[4] |
| pUA66 $P_{luxS}$ | pUA66 $P_{luxS}$-*gfpmut2*, $KAN^R$ | Zaslaver and colleagues[4] |
| pUA66 $P_{mazE}$ | pUA66 $P_{mazE}$-*gfpmut2*, $KAN^R$ | Zaslaver and colleagues[4] |

| | | |
|---|---|---|
| pUA66 $P_{mdtK}$ | pUA66 $P_{mdtK}$-*gfpmut2*, $KAN^R$ | Zaslaver and colleagues[4] |
| pUA66 $P_{menG}$ | pUA66 $P_{menG}$-*gfpmut2*, $KAN^R$ | Zaslaver and colleagues[4] |
| pUA66 $P_{mglB}$ | pUA66 $P_{mglB}$-*gfpmut2*, $KAN^R$ | Zaslaver and colleagues[4] |
| pUA66 $P_{msbB}$ | pUA66 $P_{msbB}$-*gfpmut2*, $KAN^R$ | Zaslaver and colleagues[4] |
| pUA66 $P_{mutS}$ | pUA66 $P_{mutS}$-*gfpmut2*, $KAN^R$ | Zaslaver and colleagues[4] |
| pUA66 $P_{narU}$ | pUA66 $P_{narU}$-*gfpmut2*, $KAN^R$ | Zaslaver and colleagues[4] |
| pUA66 $P_{nrdB}$ | pUA66 $P_{nrdB}$-*gfpmut2*, $KAN^R$ | Zaslaver and colleagues[4] |
| pUA66 $P_{osmB}$ | pUA66 $P_{osmB}$-*gfpmut2*, $KAN^R$ | Zaslaver and colleagues[4] |
| pUA66 $P_{osmC}$ | pUA66 $P_{osmC}$-*gfpmut2*, $KAN^R$ | Zaslaver and colleagues[4] |
| pUA66 $P_{osmE}$ | pUA66 $P_{osmE}$-*gfpmut2*, $KAN^R$ | Zaslaver and colleagues[4] |
| pUA66 $P_{otsB}$ | pUA66 $P_{otsB}$-*gfpmut2*, $KAN^R$ | Zaslaver and colleagues[4] |
| pUA66 $P_{oxyR}$ | pUA66 $P_{oxyR}$-*gfpmut2*, $KAN^R$ | Zaslaver and colleagues[4] |
| pUA66 $P_{pcnB}$ | pUA66 $P_{pcnB}$-*gfpmut2*, $KAN^R$ | Zaslaver and colleagues[4] |
| pUA66 $P_{pfkA}$ | pUA66 $P_{pfkA}$-*gfpmut2*, $KAN^R$ | Zaslaver and colleagues[4] |
| pUA66 $P_{pfkB}$ | pUA66 $P_{pfkB}$-*gfpmut2*, $KAN^R$ | Zaslaver and colleagues[4] |
| pUA66 $P_{pgmI}$ | pUA66 $P_{pgmI}$-*gfpmut2*, $KAN^R$ | Zaslaver and colleagues[4] |
| pUA66 $P_{polB}$ | pUA66 $P_{polB}$-*gfpmut2*, $KAN^R$ | Zaslaver and colleagues[4] |
| pUA66 $P_{poxB}$ | pUA66 $P_{poxB}$-*gfpmut2*, $KAN^R$ | Zaslaver and colleagues[4] |
| pUA66 $P_{pqiA}$ | pUA66 $P_{pqiA}$-*gfpmut2*, $KAN^R$ | Zaslaver and colleagues[4] |
| pUA66 $P_{pstC}$ | pUA66 $P_{pstC}$-*gfpmut2*, $KAN^R$ | Zaslaver and colleagues[4] |
| pUA66 $P_{pstS}$ | pUA66 $P_{pstS}$-*gfpmut2*, $KAN^R$ | Zaslaver and colleagues[4] |
| pUA66 $P_{recA}$ | pUA66 $P_{recA}$-*gfpmut2*, $KAN^R$ | Zaslaver and colleagues[4] |
| pUA66 $P_{recN}$ | pUA66 $P_{recN}$-*gfpmut2*, $KAN^R$ | Zaslaver and colleagues[4] |
| pUA66 $P_{relB}$ | pUA66 $P_{relB}$-*gfpmut2*, $KAN^R$ | Zaslaver and colleagues[4] |
| pUA66 $P_{rhaS}$ | pUA66 $P_{rhaS}$-*gfpmut2*, $KAN^R$ | Zaslaver and colleagues[4] |
| pUA66 $P_{rhlE}$ | pUA66 $P_{rhlE}$-*gfpmut2*, $KAN^R$ | Zaslaver and colleagues[4] |
| pUA66 $P_{rpoH}$ | pUA66 $P_{rpoH}$-*gfpmut2*, $KAN^R$ | Zaslaver and colleagues[4] |
| pUA66 $P_{rpoS}$ | pUA66 $P_{rpoS}$-*gfpmut2*, $KAN^R$ | Zaslaver and colleagues[4] |
| pUA66 $P_{rsd}$ | pUA66 $P_{rsd}$-*gfpmut2*, $KAN^R$ | Zaslaver and colleagues[4] |
| pUA66 $P_{rssA}$ | pUA66 $P_{rssA}$-*gfpmut2*, $KAN^R$ | Zaslaver and colleagues[4] |
| pUA66 $P_{rssB}$ | pUA66 $P_{rssB}$-*gfpmut2*, $KAN^R$ | Zaslaver and colleagues[4] |
| pUA66 $P_{ruvA}$ | pUA66 $P_{ruvA}$-*gfpmut2*, $KAN^R$ | Zaslaver and colleagues[4] |
| pUA66 $P_{sbmC}$ | pUA66 $P_{sbmC}$-*gfpmut2*, $KAN^R$ | Zaslaver and colleagues[4] |
| pUA66 $P_{slyD}$ | pUA66 $P_{slyD}$-*gfpmut2*, $KAN^R$ | Zaslaver and colleagues[4] |
| pUA66 $P_{sodC}$ | pUA66 $P_{sodC}$-*gfpmut2*, $KAN^R$ | Zaslaver and colleagues[4] |
| pUA66 $P_{sohA}$ | pUA66 $P_{sohA}$ -*gfpmut2*, $KAN^R$ | Zaslaver and colleagues[4] |
| pUA66 $P_{sohB}$ | pUA66 $P_{sohB}$-*gfpmut2*, $KAN^R$ | Zaslaver and colleagues[4] |
| pUA66 $P_{speB}$ | pUA66 $P_{speB}$-*gfpmut2*, $KAN^R$ | Zaslaver and colleagues[4] |
| pUA66 $P_{speC}$ | pUA66 $P_{speC}$-*gfpmut2*, $KAN^R$ | Zaslaver and colleagues[4] |
| pUA66 $P_{sulA}$ | pUA66 $P_{sulA}$-*gfpmut2*, $KAN^R$ | Zaslaver and colleagues[4] |
| pUA66 $P_{talA}$ | pUA66 $P_{talA}$-*gfpmut2*, $KAN^R$ | Zaslaver and colleagues[4] |

| | | |
|---|---|---|
| pUA66 $P_{tam}$ | pUA66 $P_{tam}$-*gfpmut2*, $KAN^R$ | Zaslaver and colleagues[4] |
| pUA66 $P_{tktB}$ | pUA66 $P_{tktB}$-*gfpmut2*, $KAN^R$ | Zaslaver and colleagues[4] |
| pUA66 $P_{tpiA}$ | pUA66 $P_{tpiA}$-*gfpmut2*, $KAN^R$ | Zaslaver and colleagues[4] |
| pUA66 $P_{treA}$ | pUA66 $P_{treA}$-*gfpmut2*, $KAN^R$ | Zaslaver and colleagues[4] |
| pUA66 $P_{treF}$ | pUA66 $P_{treF}$-*gfpmut2*, $KAN^R$ | Zaslaver and colleagues[4] |
| pUA66 $P_{umuD}$ | pUA66 $P_{umuD}$-*gfpmut2*, $KAN^R$ | Zaslaver and colleagues[4] |
| pUA66 $P_{uspB}$ | pUA66 $P_{uspB}$-*gfpmut2*, $KAN^R$ | Zaslaver and colleagues[4] |
| pUA66 $P_{uvrA}$ | pUA66 $P_{uvrA}$-*gfpmut2*, $KAN^R$ | Zaslaver and colleagues[4] |
| pUA66 $P_{uvrD}$ | pUA66 $P_{uvrD}$-*gfpmut2*, $KAN^R$ | Zaslaver and colleagues[4] |
| pUA66 $P_{wrbA}$ | pUA66 $P_{wrbA}$-*gfpmut2*, $KAN^R$ | Zaslaver and colleagues[4] |
| pUA66 $P_{xapA}$ | pUA66 $P_{xapA}$-*gfpmut2*, $KAN^R$ | Zaslaver and colleagues[4] |
| pUA66 $P_{yabI}$ | pUA66 $P_{yabI}$-*gfpmut2*, $KAN^R$ | Zaslaver and colleagues[4] |
| pUA66 $P_{yafN}$ | pUA66 $P_{yafN}$-*gfpmut2*, $KAN^R$ | Zaslaver and colleagues[4] |
| pUA66 $P_{ybfE}$ | pUA66 $P_{ybfE}$-*gfpmut2*, $KAN^R$ | Zaslaver and colleagues[4] |
| pUA66 $P_{ybgA}$ | pUA66 $P_{ybgA}$-*gfpmut2*, $KAN^R$ | Zaslaver and colleagues[4] |
| pUA66 $P_{ybjP}$ | pUA66 $P_{ybjP}$-*gfpmut2*, $KAN^R$ | Zaslaver and colleagues[4] |
| pUA66 $P_{yciG}$ | pUA66 $P_{yciG}$-*gfpmut2*, $KAN^R$ | Zaslaver and colleagues[4] |
| pUA66 $P_{yciT}$ | pUA66 $P_{yciT}$-*gfpmut2*, $KAN^R$ | Zaslaver and colleagues[4] |
| pUA66 $P_{ycjK}$ | pUA66 $P_{ycjK}$ -*gfpmut2*, $KAN^R$ | Zaslaver and colleagues[4] |
| pUA66 $P_{ycjL}$ | pUA66 $P_{ycjL}$ -*gfpmut2*, $KAN^R$ | Zaslaver and colleagues[4] |
| pUA66 $P_{ydbD}$ | pUA66 $P_{ydbD}$-*gfpmut2*, $KAN^R$ | Zaslaver and colleagues[4] |
| pUA66 $P_{ydcS}$ | pUA66 $P_{ydcS}$-*gfpmut2*, $KAN^R$ | Zaslaver and colleagues[4] |
| pUA66 $P_{yebG}$ | pUA66 $P_{yebG}$-*gfpmut2*, $KAN^R$ | Zaslaver and colleagues[4] |
| pUA66 $P_{yeeU}$ | pUA66 $P_{yeeU}$-*gfpmut2*, $KAN^R$ | Zaslaver and colleagues[4] |
| pUA66 $P_{yehZ}$ | pUA66 $P_{yehZ}$-*gfpmut2*, $KAN^R$ | Zaslaver and colleagues[4] |
| pUA66 $P_{yfjG}$ | pUA66 $P_{yfjG}$-*gfpmut2*, $KAN^R$ | Zaslaver and colleagues[4] |
| pUA66 $P_{yfjN}$ | pUA66 $P_{yfjN}$ -*gfpmut2*, $KAN^R$ | Zaslaver and colleagues[4] |
| pUA66 $P_{yggE}$ | pUA66 $P_{yggE}$-*gfpmut2*, $KAN^R$ | Zaslaver and colleagues[4] |
| pUA66 $P_{ygiU}$ | pUA66 $P_{ygiU}$ -*gfpmut2*, $KAN^R$ | Zaslaver and colleagues[4] |
| pUA66 $P_{ygjN}$ | pUA66 $P_{ygjN}$-*gfpmut2*, $KAN^R$ | Zaslaver and colleagues[4] |
| pUA66 $P_{yhaJ}$ | pUA66 $P_{yhaJ}$-*gfpmut2*, $KAN^R$ | Zaslaver and colleagues[4] |
| pUA66 $P_{yhhT}$ | pUA66 $P_{yhhT}$-*gfpmut2*, $KAN^R$ | Zaslaver and colleagues[4] |
| pUA66 $P_{yhjG}$ | pUA66 $P_{yhjG}$-*gfpmut2*, $KAN^R$ | Zaslaver and colleagues[4] |
| pUA66 $P_{yiaG}$ | pUA66 $P_{yiaG}$-*gfpmut2*, $KAN^R$ | Zaslaver and colleagues[4] |
| pUA66 $P_{yjiW}$ | pUA66 $P_{yjiW}$ -*gfpmut2*, $KAN^R$ | Zaslaver and colleagues[4] |
| pUA66 $P_{yjiY}$ | pUA66 $P_{yjiY}$-*gfpmut2*, $KAN^R$ | Zaslaver and colleagues[4] |
| pUA66 $P_{yjjK}$ | pUA66 $P_{yjjK}$-*gfpmut2*, $KAN^R$ | Zaslaver and colleagues[4] |
| pUA66 $P_{ykfI}$ | pUA66 $P_{ykfI}$-*gfpmut2*, $KAN^R$ | Zaslaver and colleagues[4] |
| pUA66 $P_{ymcE}$ | pUA66 $P_{ymcE}$-*gfpmut2*, $KAN^R$ | Zaslaver and colleagues[4] |
| pUA66 $P_{ynfE}$ | pUA66 $P_{ynfE}$-*gfpmut2*, $KAN^R$ | Zaslaver and colleagues[4] |
| pUA66 $P_{yoaA}$ | pUA66 $P_{yoaA}$-*gfpmut2*, $KAN^R$ | Zaslaver and colleagues[4] |

| | | |
|---|---|---|
| pUA66 $P_{znuA}$ | pUA66 $P_{znuA}$-*gfpmut2*, $KAN^R$ | Zaslaver and colleagues[4] |

**Supplementary Table S2. Oligonucleotides. (a)** Persister-FACSeq primers. **(b)** Oligonucleotides for mutant and plasmid construction and confirmation. **(c)** qPCR primers.

| **a. Persister-FACSeq primers** | | |
|---|---|---|
| **FACS Quantile** | **Untreated or Treated Sample** | **Forward Primer [a b] (5' → 3')** |
| A | Untreated | *AATGATACGGCGACCACCGAGATCTACACTCTTTCCCTACACGACGCTCTTCCGATCT***TTAGGC**TATCACGAGGCCCTTTCGTC |
| A | Treated | *AATGATACGGCGACCACCGAGATCTACACTCTTTCCCTACACGACGCTCTTCCGATCT***GGCTAC**TATCACGAGGCCCTTTCGTC |
| B | Untreated | *AATGATACGGCGACCACCGAGATCTACACTCTTTCCCTACACGACGCTCTTCCGATCT***TGACCA**TATCACGAGGCCCTTTCGTC |
| B | Treated | *AATGATACGGCGACCACCGAGATCTACACTCTTTCCCTACACGACGCTCTTCCGATCT***CTTGTA**TATCACGAGGCCCTTTCGTC |
| C | Untreated | *AATGATACGGCGACCACCGAGATCTACACTCTTTCCCTACACGACGCTCTTCCGATCT***ACAGTG**TATCACGAGGCCCTTTCGTC |
| C | Treated | *AATGATACGGCGACCACCGAGATCTACACTCTTTCCCTACACGACGCTCTTCCGATCT***AGTCAA**TATCACGAGGCCCTTTCGTC |
| D | Untreated | *AATGATACGGCGACCACCGAGATCTACACTCTTTCCCTACACGACGCTCTTCCGATCT***GCCAAT**TATCACGAGGCCCTTTCGTC |
| D | Treated | *AATGATACGGCGACCACCGAGATCTACACTCTTTCCCTACACGACGCTCTTCCGATCT***AGTTCC**TATCACGAGGCCCTTTCGTC |
| E | Untreated | *AATGATACGGCGACCACCGAGATCTACACTCTTTCCCTACACGACGCTCTTCCGATCT***CAGATC**TATCACGAGGCCCTTTCGTC |
| E | Treated | *AATGATACGGCGACCACCGAGATCTACACTCTTTCCCTACACGACGCTCTTCCGATCT***ATGTCA**TATCACGAGGCCCTTTCGTC |
| F | Untreated | *AATGATACGGCGACCACCGAGATCTACACTCTTTCCCTACACGACGCTCTTCCGATCT***TAGCTT**TATCACGAGGCCCTTTCGTC |
| F | Treated | *AATGATACGGCGACCACCGAGATCTACACTCTTTCCCTACACGACGCTCTTCCGATCT***CCGTCC**TATCACGAGGCCCTTTCGTC |

[a] Illumina adapter is in italics. Quantile-specific barcode is in bold. The remainder of the sequence (20nts) is homologous to the plasmid sequence 5bps upstream of the XhoI cut site which was used for promoter cloning[4].

[b] Reverse primer for all samples was 5' – *CAAGCAGAAGACGGCATACGAGAT*TCCTTTACTCATATGTATATCTCCTTCTT– 3'. The italicized region is the Illumina adapter. The remainder of the sequence (29nts) is homologous to

the pUA66 plasmid sequence 9bps downstream from the BamHI cut site which was used for promoter cloning[4].

| **b. Oligonucleotides for mutant and plasmid construction and confirmation** | | |
|---|---|---|
| **Oligonucleotides for mutant construction** | | |
| **Strain** | **Forward (5' → 3')** | **Reverse (5' → 3')** |
| Δ*lacZ*::KAN$^{R}$ ***a b*** | GGAATTGTGAGCGGATAACAATTTCACACAGGAAACAGCTGAGCGATTGTGTAGGCTGGA | TTACGCGAAATACGGGCAGACATGGCCTGCCCGGTTATTATTAACGGCTGACATGGGAAT |
| Δ*lacI*::KAN$^{R}$ ***b c*** | GGGATCAGGAGGAGAAGATCG | TTATCCGCTCACAATTCCAC |
| *attB*::lacI$^{q}$ GENT$^{R}$ Set 1 ***d*** | CTATGAAATAGAAAAATGAATCCGTTGAAGCCTGCTTTTTGACACCATCGAATGGTGCAA | CTGCTGCGTAACATCGTTGCTTCTCCTTCTTAAATCTAGATCACTGCCCGCTTTCCAGTC |
| *attB*::lacI$^{q}$ GENT$^{R}$ Set 2 ***d*** | AGCAACGATGTTACGCAGCA | TTTTGTCTTTTTACCTTCCCGTTTCGCTCAAGTTAGTATATTAGGTGGCGGTACTTGGGT |
| Δ*rssB::KanR*$^{a\ b}$ | ATGCCACTATTGAGTAAAGCCAGTCAGGGGAGAGAACATGGTGTAGGCTGGAGCTGCTTC | AGCCCGCGTTATCGTTTGCTCATTCTGCAGACAACATCAACATATGAATATCCTCCTTAG |
| **Oligonucleotides for Plasmid Construction** | | |
| **Strain** | **Forward (5' → 3')** | **Reverse (5' → 3')** |
| P$_{dinD}$-*gfpmut2* (pTH01) | GCATCATGCTAGCACTCGTAACG | ATCATCACTCGAGTGCCCACTC |
| P$_{T5}$-*mCherry* (pTH03) | GCGCGGAGCGCTTCCTTTTAAATTAAAAATGA | GCGCGGGACGTCAGGTGGCACTTTTCGGGGAA |
| P$_{T5}$-*rpoS* (pTH04) | TCATCATCCGGAATTCATTAAAGAGGAGGAATTAACTATGAGTCAGAATACGCTGAAAGT | TCATCATCGAAGCTTTTACTCGCGGAACAGCGCTT |
| P$_{T5}$-*bolA* (pTH05) | TCATCATCCGGAATTCATTAAAGAGGAGGAATTAACTATGATGATACGTGAGCGGATAGA | TCATCATCGAAGCTTTTACGCGATGCTTCCTGCTC |
| P$_{T5}$-*csiE* (pTH06) | TCATCATCCGGAATTCATTAAAGAGGAGGAATTAACTATGATGCCTACGCTTGCTCCACC | TCATCATCGAAGCTTTCATGCTGATTCGAGCATTT |
| P$_{T5}$-*rssB* (pTH07) | TCATCATCCGGAATTCATTAAAGAGGAGGAATTAACTATGACGCAGCCATTGGTCGGAAA | TCATCATCGAAGCTTTCATTCTGCAGACAACATCA |
| P$_{T5}$-*csiD-lhgO-gabDTP* (pTH08) | TCATCATCCGGAATTCATTAAAGAGGAGGAATTAACTATGAATGCACTGACCGCCGTACA | TCATCATCGAAGCTTTCAGCGCGTATTATGAACGG |
| P$_{T5}$-*ydcSTUV-patD* (pTH09) | TCATCATCCGGAATTCATTAAAGAGGAGGAATTAACTATGAGCAAGACATTTGCCCGCAG | TCATCATCGGGATCCTTAATGTTTAACCATGACGT |

| Oligonucleotides to Confirm Genetic Mutants | | | | |
|---|---|---|---|---|
| **Strain** | **Upstream Forward Primer (5'→ 3')** | **Downstream Reverse Primer (5' → 3')** | **Internal Forward Primer (5' → 3')** | **Internal Reverse Primer (5' → 3')** |
| Δ*lacI*::KAN$^{R}$ [e] | GGGATCAGGAGGAGAAGATCG | TTATCCGCTCACAATTCCAC | ATCGAATGGCGCAAAACCT | TTCCAGTCGGGAAACCTGT |
| Δ*lacZ*::KAN$^{R}$ | TTTATGCTTCCGGCTCGTAT | ATTGTAACAGTGGCCCGAAG | AGGACAGTCGTTTGCCGTCT | ATCGACAGATTTGATCCAGCG |
| *attB*::lacI$^{q}$ GENT$^{R}$ | TCGAAATTCTGCGCTTCTTT | GCCTCGATTACTGCGATGTT | ATCGAATGGTGCAAAACCT | TTCCAGTCGGGAAACCTGT |
| Δ*rpoS*::KAN$^{R}$ [b] | CCATAACGACACAATGCTGGTC | TATCTGGGGTTGTCGGTAGC | AATGAAGATGCGGAATTTGATG | GGTATCTTCCGGACCGTTCTC |
| Δ*rssB*::KAN$^{R}$ [b] | TGGCAGTGGAAAGGAAAATG | GGCAACATCCTGGTTCCTAA | TATTTCGCTCGCTTCTGGAT | GTGCCTAACGGAACACCATT |
| Δ*csiE*::KAN$^{R}$ | GCTTGCCAACATTTCTGATG | | TCAGCGAGCTTAATGGTGTG | GATGGACGCCGTATTCACTT |

| Oligonucleotides to confirm plasmid construction | | |
|---|---|---|
| **Plasmid** | **Forward (5' → 3')** | **Reverse (5' → 3')** |
| $P_{dinD}$-*gfpmut2* (pTH01) | GTGCCCAGTCATAGCCGAATAGC | TCTGGGTATCTCGCAAAGCA |
| $P_{T5}$-*mCherry* (pTH03) [f] Set 1 [g] | CTTGATCCGGCAAACAAACC | GCTATGTGGCGCGGTATTAT |
| $P_{T5}$-*mCherry* (pTH03) [f] Set 2 [h] | TATTTGCTTTGTGAGCGGATA | GCTAGCTTGGATTCTCACCA |
| $P_{T5}$-*rpoS* (pTH04), $P_{T5}$-*bolA* (pTH05), $P_{T5}$-*csiE* (pTH06), $P_{T5}$-*rssB* (pTH07) $P_{T5}$-*csiD-lhgO-gabDTP* (pTH08) $P_{T5}$-*ydcSTUV-patD* (pTH09) | TATTTGCTTTGTGAGCGGATA | GCTAGCTTGGATTCTCACCA |
| $P_{T5}$-*csiD-lhgO-gabDTP* (pTH08) | GAGATAACCGATTACGTGCTGATG | |
| $P_{T5}$-*csiD-lhgO-gabDTP* (pTH08) | TCCATGCCGGGGTCTATTAC | |
| $P_{T5}$-*csiD-lhgO-gabDTP* (pTH08) | TTTGGGCGTTCATCTCACC | |
| $P_{T5}$-*csiD-lhgO-gabDTP* (pTH08) | ACAGTAACTTATTCCGCCAGCA | |
| $P_{T5}$-*csiD-lhgO-gabDTP* (pTH08) | AAACTGTCGTTTACCGGTTCG | |

| | | |
|---|---|---|
| $P_{T5}$-*csiD-lhgO-gabDTP* (pTH08) | GCTGGAGTACGGCATCGTC | |
| $P_{T5}$-*csiD-lhgO-gabDTP* (pTH08) | ATTACACGCTGGCGCTGA | |
| $P_{T5}$-*csiD-lhgO-gabDTP* (pTH08) | CCGAAAAACACCCGGAGA | |
| $P_{T5}$-*csiD-lhgO-gabDTP* (pTH08) | CAAGTCTGTTTGTCGGTTCCAG | |
| $P_{T5}$-*csiD-lhgO-gabDTP* (pTH08) | CGGTTATCTGGCGTATTTCTATCTT | |
| $P_{T5}$-*csiD-lhgO-gabDTP* (pTH08) | | GTCACACAGAGCGCGTAAAC |
| $P_{T5}$-*ydcSTUV-patD* (pTH09) | AGCCACTCAGCCGCAGTT | |
| $P_{T5}$-*ydcSTUV-patD* (pTH09) | TGGACGCAGGATTACATTGC | |
| $P_{T5}$-*ydcSTUV-patD* (pTH09) | CGGTATCACTTTTATCTTCGTTACCC | |
| $P_{T5}$-*ydcSTUV-patD* (pTH09) | GGCTCGCTGCTGACACTG | |
| $P_{T5}$-*ydcSTUV-patD* (pTH09) | CTATCTTTACCTTCTCACTCACACTGG | |
| $P_{T5}$-*ydcSTUV-patD* (pTH09) | CATCGTGGTCGGTCATGC | |
| $P_{T5}$-*ydcSTUV-patD* (pTH09) | GTGCGTGCGGAATGTCTG | |

Internal $KAN^R$ cassette reverse primer for confirmation of genetic mutants (5' → 3'):
ATGATGGATACTTTCTCGGCAGGAG

***a*** Primers consist of approximately 40nt homology regions for target locations in chromosome and approximately 20nt sequence to amplify $KAN^R$ cassette from pKD4[8].
***b*** $KAN^R$ was removed (cured) using pCP20[8].
***c*** Δ*lacI*::$KAN^R$ was amplified from Δ*lacI*::$KAN^R$ strain created for a previous work[9], and transferred into MG1655Δ*lacZ* using the method of Datsenko and Wanner[8].
***d*** Overlap extension PCR was performed to create genetic construct. Primer Set 1 forward primer contains 40nt homology to target *attB* region in chromosome and 20nt sequence to amplify $lacI^q$. Primer Set 1 reverse primer contains 20nt sequence to amplify *lacI* open reading frame, 13nt spacer region, a 6nt ribosome binding site, and 21nts to amplify gentamycin resistance gene cassette[10]. Primer Set 2 forward primer amplifies gentamycin resistance gene and is complementary to a region of the Set 1 reverse primer. Primer Set 2 reverse primer contains 20nt homology to amplify gentamycin resistance gene and 40bps homologous to *attB* region in chromosome.
***e*** Internal forward primer is internal to deletion, although it is external to *lacI*.
***f*** Construct was confirmed by phenotypic tests of ampicillin resistance and IPTG-inducible mCherry production as well as cPCR with the primers listed.
***g*** External forward primer on pQE80 backbone, internal reverse primer in the ampicillin (AMP) resistance gene.
***h*** Forward and reverse primers external to *mCherry*.

| c. | qPCR primers | |
|---|---|---|
| **Target** | **Forward (5' → 3')** | **Reverse (5' → 3')** |
| ***ydcS*** | CGAACCGCCTACCAATTTAG | GACCATTTCATCGGAAGTCG |
| ***patD*** | GCGGATATTGAAGCAGTGGT | TCCAAGCTCCGTAGACTCGT |
| ***rrsA*** | GAAGAAGCACCGGCTAACTC | TGCCAGTATCAGATGCAGTTC |

**Supplementary Table S3. Read and promoter abundances from Persister-FACSeq.**

| Persister-FACSeq Run 1 | | |
|---|---|---|
| **Barcode** | **# of reads with barcode** | **# of reads with barcode that mapped to promoters** |
| **Untreated A** | 1,089,400 | 727,048 |
| **Untreated B** | 808,922 | 529,406 |
| **Untreated C** | 996,319 | 645,071 |
| **Untreated D** | 743,126 | 466,215 |
| **Untreated E** | 788,840 | 471,475 |
| **Untreated F** | 700,866 | 536,193 |
| **Treated A** | 755,965 | 486,432 |
| **Treated B** | 1,126,379 | 749,591 |
| **Treated C** | 944,045 | 604,483 |
| **Treated D** | 837,799 | 524,174 |
| **Treated E** | 897,845 | 545,445 |
| **Treated F** | 843,871 | 716,725 |
| **Total** | 10,533,377 | 7,002,258 |

| Persister-FACSeq Run 2 | | |
|---|---|---|
| **Barcode** | **# of reads with barcode** | **# of reads with barcode that mapped to promoters** |
| **Untreated A** | 1,525,215 | 925,107 |
| **Untreated B** | 1,561,371 | 908,174 |
| **Untreated C** | 1,526,034 | 918,295 |
| **Untreated D** | 1,598,190 | 954,813 |
| **Untreated E** | 1,257,925 | 743,408 |
| **Untreated F** | 1,264,380 | 952,398 |
| **Treated A** | 1,456,711 | 884,009 |
| **Treated B** | 1,702,928 | 998,835 |
| **Treated C** | 1,442,428 | 871,825 |
| **Treated D** | 1,438,324 | 828,005 |
| **Treated E** | 1,368,152 | 809,977 |
| **Treated F** | 1,608,704 | 1,458,985 |
| **Total** | 17,750,362 | 11,253,831 |

| Persister-FACSeq Run 3 | | |
|---|---|---|
| **Barcode** | **# of reads with barcode** | **# of reads with barcode that mapped to promoters** |
| **Untreated A** | 803,943 | 527,046 |
| **Untreated B** | 904,026 | 574,324 |
| **Untreated C** | 948,495 | 612,721 |
| **Untreated D** | 887,610 | 565,896 |
| **Untreated E** | 1,083,818 | 717,814 |
| **Untreated F** | 931,560 | 652,403 |
| **Treated A** | 930,795 | 635,604 |
| **Treated B** | 1,026,828 | 700,479 |
| **Treated C** | 906,378 | 598,799 |
| **Treated D** | 919,305 | 599,786 |
| **Treated E** | 884,171 | 579,019 |
| **Treated F** | 1,064,121 | 884,144 |
| **Total** | 11,291,050 | 7,648,035 |

**Supplementary Table S4. Promoter names, mean Euclidean distances, coefficients of variation (COV), and p-values corresponding to each index in Fig. 3b.** Green background indicates promoter identified by Persister-FACSeq to have distinct normal cell and persister gene expression patterns.

| Index | Promoter | Mean Euclidean Distance | COV | p-value |
|---|---|---|---|---|
| 1 | lacZ | 0.573156542 | 0.43903 | 0.04837 |
| 2 | csiE | 0.422849974 | 0.26843 | 0.02085 |
| 3 | pfkB | 0.39101906 | 0.79318 | 0.15432 |
| 4 | bolA | 0.371245198 | 0.17088 | 0.00613 |
| 5 | rssB | 0.334677596 | 0.23317 | 0.01916 |
| 6 | uspB | 0.33169389 | 0.26488 | 0.02946 |
| 7 | ydcS | 0.30358852 | 0.25763 | 0.03034 |
| 8 | yehZ (osmF) | 0.276235562 | 0.29103 | 0.04574 |
| 9 | csiD | 0.268050348 | 0.0462 | 0.00798 |
| 10 | aldB | 0.253860383 | 0.41817 | 0.10466 |
| 11 | fadL | 0.248930736 | 0.30938 | 0.06846 |
| 12 | osmC | 0.22592144 | 0.35782 | 0.10919 |
| 13 | ygjN (higB) | 0.215443594 | 0.45113 | 0.16263 |
| 14 | dinD | 0.209289678 | 0.15265 | 0.0338 |
| 15 | umuD | 0.208579257 | 0.71179 | 0.26066 |
| 16 | ecpD (yadV) | 0.202865709 | 0.56789 | 0.2413 |
| 17 | yiaG | 0.19441949 | 0.54949 | 0.2555 |
| 18 | ykfI | 0.192017389 | 0.35252 | 0.17321 |
| 19 | galE | 0.188101242 | 0.25831 | 0.13705 |
| 20 | poxB | 0.185822235 | 0.47322 | 0.26948 |
| 21 | ydbD | 0.184078442 | 0.31538 | 0.17804 |
| 22 | rpoS | 0.182147812 | 0.46066 | 0.26885 |
| 23 | pfkA | 0.179473499 | 0.43748 | 0.24596 |
| 24 | yjiY | 0.177880444 | 0.11829 | 0.16014 |
| 25 | lexA | 0.176171224 | 0.35491 | 0.22532 |
| 26 | katE | 0.17541735 | 0.26543 | 0.21463 |
| 27 | rhlE | 0.173753003 | 0.14299 | 0.11952 |
| 28 | sbmC | 0.172725821 | 0.38807 | 0.27786 |
| 29 | gyrB | 0.171423018 | 0.44972 | 0.32361 |
| 30 | tam | 0.171404242 | 0.30097 | 0.23619 |
| 31 | tpiA | 0.171150786 | 0.57065 | 0.3528 |
| 32 | mutS | 0.17062493 | 0.23665 | 0.22705 |
| 33 | osmB | 0.169308541 | 0.66122 | 0.4137 |
| 34 | ada | 0.165745457 | 0.31586 | 0.26898 |
| 35 | hofM | 0.164419686 | 0.37839 | 0.32363 |
| 36 | ilvY | 0.161796377 | 0.37265 | 0.32075 |

| | | | | |
|---|---|---|---|---|
| **37** | **dkgB** | 0.159255116 | 0.52231 | 0.40237 |
| **38** | **tisB** | 0.158047337 | 0.44838 | 0.3785 |
| **39** | **dinG** | 0.15612918 | 0.08987 | 0.3271 |
| **40** | **dinB** | 0.154950728 | 0.59339 | 0.44421 |
| **41** | **pstS** | 0.153762631 | 0.21303 | 0.4214 |
| **42** | **hrpB** | 0.153740404 | 0.12226 | 0.37611 |
| **43** | **uvrD** | 0.153402231 | 0.13565 | 0.36117 |
| **44** | **yafN** | 0.153097395 | 0.48239 | 0.41958 |
| **45** | **rssA** | 0.152477208 | 0.32963 | 0.43788 |
| **46** | **dhaR** | 0.150672234 | 0.30493 | 0.42339 |
| **47** | **yhhT** | 0.149283707 | 0.44309 | 0.45233 |
| **48** | **ybgA** | 0.1491488 | 0.74308 | 0.4786 |
| **49** | **mdtK** | 0.146655201 | 0.51604 | 0.48303 |
| **50** | **recA** | 0.145924298 | 0.53109 | 0.47271 |
| **51** | **yhjG** | 0.145884614 | 0.36553 | 0.492 |
| **52** | **cbdA (appC)** | 0.14535587 | 0.46186 | 0.49762 |
| **53** | **ftsK(dinH)** | 0.144602142 | 0.38788 | 0.4752 |
| **54** | **ycjK (puuA)** | 0.144310683 | 0.44545 | 0.49505 |
| **55** | **ego (lsrA)** | 0.143694958 | 0.47829 | 0.48806 |
| **56** | **xapA** | 0.141531823 | 0.17994 | 0.38575 |
| **57** | **asr** | 0.141001987 | 0.51367 | 0.4941 |
| **58** | **oxyR** | 0.140645867 | 0.29247 | 0.47957 |
| **59** | **ruvA** | 0.138564473 | 0.12234 | 0.29218 |
| **60** | **b0257** | 0.137331239 | 0.45227 | 0.4406 |
| **61** | **yciT** | 0.135225883 | 0.30948 | 0.40818 |
| **62** | **blc** | 0.13378457 | 0.67476 | 0.43536 |
| **63** | **nrdB** | 0.132802684 | 0.05538 | 0.33685 |
| **64** | **tktB** | 0.131785984 | 0.13732 | 0.34004 |
| **65** | **pqiA** | 0.131737717 | 0.12128 | 0.30567 |
| **66** | **hdeA** | 0.131173097 | 0.09074 | 0.30449 |
| **67** | **hdfR** | 0.130822037 | 0.26137 | 0.31246 |
| **68** | **hipB** | 0.130428872 | 0.886 | 0.42288 |
| **69** | **ynfE** | 0.129950969 | 0.6629 | 0.4173 |
| **70** | **insN-1 (b0255)** | 0.129625592 | 0.3788 | 0.32571 |
| **71** | **sohA(prlF)** | 0.129171389 | 0.37124 | 0.31888 |
| **72** | **frdA** | 0.128789858 | 0.66692 | 0.40693 |
| **73** | **glgS** | 0.122542098 | 0.52062 | 0.29049 |
| **74** | **cfa** | 0.119979358 | 0.4752 | 0.27743 |
| **75** | **rhaS** | 0.118830047 | 0.60525 | 0.29754 |

| | | | | |
|---|---|---|---|---|
| **76** | **adrA** | 0.114448034 | 0.61881 | 0.286 |
| **77** | **polB (dinA)** | 0.112293949 | 0.18718 | 0.12267 |
| **78** | **dacB** | 0.109809328 | 0.19963 | 0.08093 |
| **79** | **pgmI (gpmC)** | 0.10952029 | 0.28897 | 0.12048 |
| **80** | **yabI** | 0.109292458 | 0.3918 | 0.16045 |
| **81** | **yeeU** | 0.09982288 | 0.42224 | 0.1015 |
| **82** | **eno** | 0.09797189 | 0.72877 | 0.20218 |
| **83** | **pcnB** | 0.092791621 | 1.11839 | 0.24126 |
| **84** | **artP** | 0.09054561 | 1.01551 | 0.20669 |
| **85** | **ycjL (puuD)** | 0.088476962 | 1.18444 | 0.22962 |
| **86** | **sohB** | 0.086886419 | 0.41413 | 0.06253 |
| **87** | **ihfB** | 0.081893643 | 0.73617 | 0.08926 |
| **88** | **rsd** | 0.081134182 | 1.56648 | 0.23429 |
| **89** | **fliY** | 0.073460976 | 1.51818 | 0.18625 |
| **90** | **rpoH** | 0.066934973 | 1.68561 | 0.18986 |
| **91** | **mglB** | 0.048832376 | 1.64747 | 0.08001 |

## SUPPLEMENTARY METHODS

### Bacterial strains

Strain TH001 is Δ*lacI*Δ*lacZ* and strain TH002 is an $OFL^R$ mutant derived from TH001. Strain TH002 was derived by growing strain TH001 bearing the $P_{lacZ}$-*gfp* reporter plasmid, which contained a kanamycin resistance cassette for selection, in LB medium containing 50µg/mL KAN in test tubes for 16 h O/N at 37°C with shaking (250rpm), followed by plating approximately $10^9$ cells on LB agar plates containing KAN (50µg/mL) and OFL at 2x, 4x, or 8x the MIC. MIC of OFL for wild-type MG1655 was determined by serial 2-fold dilutions of OFL in LB broth[11] and was found to be 0.039-0.078µg/mL. Plates were incubated for 24-48 h at 37°C. All CFUs were then harvested from the plate with the highest OFL concentration that grew >2 colonies using LB medium containing 50µg/mL KAN. Cultures were then grown 16 h at 37°C with shaking, after which, fluorescence of the culture was measured to ensure that the $P_{lacZ}$-*gfp* reporter plasmid was still functioning and approximately $10^9$ cells were again plated on LB agar plates containing 50µg/mL KAN and 2x, 4x, or 8x the OFL concentration from which the cells were harvested. This process was repeated until colonies grew on plates containing 5µg/mL OFL (the concentration of OFL used for antibiotic tolerance assays in this work). Colonies were streaked on LB agar plates containing 50µg/mL KAN and 5µg/mL OFL and then individual colonies were grown in LB media containing 50µg/mL KAN and 5µg/mL OFL. Fluorescence and a Δ*lacZ* phenotype (white colonies on plates containing IPTG and Xgal) were confirmed, growth assays in 5µg/mL OFL confirmed resistance (Supplementary Fig. S3), and antibiotic tolerance assays were conducted to ensure high survival after 5 h of treatment with 5µg/mL OFL in stationary phase. Strain TH003 was raised identically to TH002. Strain TH004, which is Δ*lacI*Δ*lacZ attB*::$lacI^q$ $GENT^R$, was derived by inserting the $lacI^q$ promoter, *lacI* open reading frame (ORF), and a gentamycin resistance cassette[10] into the *attB* site of TH001.

Genetic mutations were either performed using the method of Datsenko and Wanner[8] using the primers listed in Supplementary Table S2 or transduced from the Keio collection[3] using the standard P1 phage method. The kanamycin resistance marker was removed from specified strains in Supplementary Table S2b with FLP recombinase[8]. All mutations were confirmed using PCR and/or DNA sequencing (Genewiz, South Plainfield, NJ) using the primers listed in Supplementary Table S2b.

### Plasmid Constructions

Enzymes for plasmid constructions were purchased from New England BioLabs, Inc. Restriction digests and ligations were performed according to manufacturer's protocols.

Plasmid pTH01 containing $P_{dinD}$-*gfp* was constructed by inserting the *dinD* promoter in the NheI and Xho1 cut sites of pKV000[5], a version of pUA66[4] containing an NheI cut site, using the primers listed in Supplementary Table S2b. Plasmid pTH03 containing $P_{T5}$-*mCherry* on the pQE80 $AMP^R$ backbone was constructed by inserting $AMP^R$ in place of the $KAN^R$ region of pTH02, a plasmid previously constructed that contained $P_{T5}$-*mCherry* on a pQE80 $KAN^R$ backbone[7]. The primers listed in Supplementary Table S2b were used to amplify the $AMP^R$ region of pQE80 (QIAGEN) and append it with AfeI and AatII cut sites. AfeI and AatII restriction enzymes were used to remove $KAN^R$ from pTH02 and replace it with $AMP^R$. Plasmids pTH04 ($P_{T5}$-*rpoS*), pTH05 ($P_{T5}$-*bolA*), pTH06 ($P_{T5}$-*csiE*), pTH07 ($P_{T5}$-*rssB*), and pTH08 ($P_{T5}$-*csiD-lhgO-gabDTP*) were constructed by cloning the ORF of each gene or the entire operon sequence into the EcoRI and HindIII restriction enzyme sites of pQE80 ($AMP^R$) using the primers listed in Supplementary Table S2b. pTH09 ($P_{T5}$-*ydcSTUV-patD*) was constructed by cloning the operon into the EcoRI and BamHI restriction enzyme sites of pQE80 ($AMP^R$) using the primers listed in Supplementary Table S2b (BamHI was used instead of HindIII because an internal HindIII restriction site is present in the *ydcSTUV-patD* operon). Plasmid constructs were confirmed by cPCR and/or sequencing using the primers in Supplementary Table S2b.

### Chemicals, media, and growth conditions

All chemicals were purchased from Fisher Scientific or Sigma-Aldrich unless stated otherwise. Isopropyl-β-D-thiogalactopyranoside (IPTG) was purchased from Gold Biotechnology (St. Louis, MO). AccuCount fluorescent particles for cell counting were purchased from Spherotech, Inc (Lake Forest, IL). Breathe-Easy® film was purchased from USA Scientific, Inc (Oscala, FL). Certified Molecular Biology Agarose, Laemmli Sample Buffer, 12% Mini-PROTEAN® TGX™ precast polyacrylamide gels, and Coomassie Brilliant Blue were purchased from Bio-Rad (Hercules, CA). LB medium and LB agar plates were used for all experiments; KAN was added to media and plates for plasmid retention. For selection, 50µg/mL KAN, 15µg/mL GENT, and 100µg/mL AMP were used. For antibiotic tolerance assays, OFL was used at 5µg/mL. For blue/white screening, 1mM IPTG and 40µg/mL Xgal were added to LB agar plates. For FACS, antibiotic tolerance assays, and flow cytometry, overnight cultures were prepared by culturing cells from a 25% glycerol, -80° stock in 25mL LB medium, with 50µg/mL KAN and/or 100µg/mL AMP for plasmid retention as needed, in a 250mL flask at 37°C with shaking (250rpm) for 16 h. For growth assays of $OFL^R$ strains and their parent (Supplementary Fig. S3), a test tube containing 2mL LB with

50µg/mL KAN was inoculated with a scraping from the appropriate 25% glycerol, -80°C stock and grown 16 h at 37°C with shaking (250 rpm). Cultures were diluted to $OD_{600}$~0.01 in a test tube containing 2mL LB with 50µg/mL KAN and incubated with shaking (250 rpm) at 37°C. At t=2 h, cultures were treated with 5µg/mL OFL or the equivalent volume of sterile deionized (dI) $H_2O$. $OD_{600}$ was monitored hourly for 8 h.

**Staining with SYTO9 and PI for LIVE/DEAD assays**

SYTO9 and propidium iodide (PI) are nucleic acid stains which differ in their ability to penetrate cell membranes. SYTO9, a green fluorescent dye, stains all cells, whereas PI only penetrates bacteria with compromised membranes (qualified as dead). Therefore, live cells are identified as those which fluoresce green and lack red fluorescence, whereas dead cells are those which fluoresce red.

Samples of MG1655 bearing the empty pUA66 vector were treated with OFL following the standard treatment procedure for antibiotic tolerance assays. Briefly, samples were treated for 5 hours with 5µg/mL OFL in 50% spent media/50% PBS at the cell density necessitated by FACS (~200,000 cells/mL). At the completion of treatment, samples were centrifuged and washed with 0.85% NaCl in dI $H_2O$, as per the manufacturer's protocol for SYTO9/PI staining. After removal of OFL, the samples were resuspended in 0.85% NaCl and concentrated ~10-fold to a final density of ~2,000,000 cells/mL. Concentration minimized background noise during flow cytometry (which can result from electronic noise or small particles in 0.85% NaCl). The sample was stained with PI and SYTO9 according to the manufacturer's instructions. Sphero$^{TM}$ AccuCount Fluorescent Particles were then added to the sample to facilitate cell counting, and flow cytometry was performed. As a dead-cell control, 10µl of an overnight sample of MG1655 bearing the empty pUA66 vector were incubated in 1mL of 70% ethanol for 1 h with shaking approximately every 15 min, then washed with a 0.85% NaCl solution and either left unstained or stained with PI and/or SYTO9. As a live-cell control, 10µL of an overnight sample of MG1655 bearing the empty pUA66 vector were diluted into 1mL of 0.85% NaCl solution and either left unstained or stained with PI and/or SYTO9. These dead- and live-cell controls were used to determine the gates of dead- and live-cell populations for flow cytometry.

**Flow cytometry**

A LSRII flow cytometer (BD Biosciences, San Jose, CA) was used to analyze samples. Forward and side scatter parameters (FSC and SSC) were used to identify microorganisms. Lasers emitting at 488nm and 561nm were used for excitation of GFP and mCherry, respectively. Fluorescence intensities were collected with 525/50nm and 610/20nm band-pass filters for GFP and mCherry, respectively. For LIVE/DEAD assays, a laser emitting at 488nm was used for excitation of both SYTO9 and PI, and fluorescence intensities were collected with 525/50nm and 610/20nm band-pass filters, respectively. FACSDiVa software (BD Biosciences, San Jose, CA) was used for data acquisition and FlowJo (TreeStar, Ashland, OR) software was used for data analysis.

**Fluorescence-activated cell sorting (FACS)**

Prior to cell sorting, samples were diluted to an OD~0.03-0.08 in sterile-filtered spent media to prevent clogging of the cell sorter. All sorting experiments were performed using the FACSVantage SE w/DiVa cell sorter with FACSDiVa software (BD Biosciences, San Jose, CA), at a pressure of 16 lb/in$^2$ with a 70-µm nozzle. Forward and side scatter parameters (FSC and SSC) were used to identify microorganisms. GFP-positive cells were identified by measuring green fluorescence (488nm excitation and a 530/30 bandpass filter). Cells were sorted using sterile PBS as sheath fluid and were sorted at room temperature into 5mL polystyrene round-bottom tubes. If antibiotic tolerance assays were to be performed on the samples, an equivalent volume of sterile-filtered spent media from the culture was added to the sample and KAN was added to the PBS if plasmid retention was needed, resulting in cells suspended in a 50/50 mix of PBS/spent media with 50µg/mL KAN.

**FACS reanalysis**

To ensure the accuracy of FACS sorting, 100k-200k events were collected from quantiles A and D using the FACSVantage SE w/DiVa cell sorter, and 50k events were reanalyzed on the LSRII flow cytometer (Supplementary Fig. S6b). Because sorted samples are resuspended in PBS at a very low cell density, sorted samples analyzed on the flow cytometer can have substantially more background events ("noise") than the original samples. Therefore, a sample of PBS that had passed through the cell sorter was also collected and analyzed on the LSRII cytometer. FlowJo (TreeStar, Ashland, OR) software was used to identify PBS noise based on FSC and SSC and gate out >90% of this noise, leaving ≥94% of events of the original promoter reporter sample. FlowJo software was then used to determine the mean fluorescence of each of the samples.

**Hierarchical clustering analysis**

Differences between persister proportions and normal cell proportions (***Pprop*** and ***Nprop***) for each promoter identified to have distinct normal cell and persister gene expression patterns were generated into a heat map using Matlab (R2014b) and hierarchical clustering was performed using the nearest neighbor method and a Euclidean distance metric (Matlab, R2014b).

**Confirmation of promoter sequences of reporters tested in monoculture**

Promoter reporters identified by HT sequencing results as likely to have distinct normal cell and persister gene expression patterns were selected and their identity confirmed by sequencing (Genewiz, South Plainfield, NJ). Transcriptional reporters were then subjected to monoculture FACS and OFL tolerance assays following the procedures described above.

**Statistical analysis of Persister-FACSeq predictions**

The power of Persister-FACSeq was assessed using the null hypothesis that random selection of reporters would perform as well as our method at distinguishing between promoters that exhibited distinct normal cell and persister gene expression patterns and those that did not. In total, we assayed 8 reporter strains for distinct normal cell and persister gene expression patterns, and 6 (*N*) of those exhibited different persister and normal cell distributions, whereas 2 (*M*) exhibited the same persister and normal cell distributions. We calculated the total number of ways to pick 6 (or 2) from 8, which is $\binom{8}{6} = \binom{8}{2} = 28$. Persister-FACSeq was perfectly accurate in its assessment of promoters with distinct normal cell and persister gene expression patterns (all 8 correctly identified) and that corresponded to only 1 of the 28 possible ways to pick 6 (or 2) from 8, yielding a probability that random selection would perform equitably to Persister-FACSeq of 0.036. In a more general form, the probability that random selection would perform equally or better than Persister-FACSeq can be calculated from the formula,

$$\frac{\sum_{i=0,j}\binom{M}{i}\binom{N}{i}}{\binom{N+M}{N}} \quad \text{(S1)},$$

where *j* is the number of incorrect assignments.

**Microscopy**

To visualize the morphology of cells overexpressing *bolA*, MG1655 bearing $P_{T5}$-*bolA* or $P_{T5}$-*mCherry* (control) was inoculated from a 25% glycerol, -80°C stock into a test tube containing 2mL LB with 100µg/mL AMP and incubated at 37°C with shaking (250rpm). IPTG was added at t=12 h for a final concentration of 1mM IPTG. Samples were incubated four more hours at 37°C and 250rpm. At t=16 h, samples were centrifuged for 3 min at 15,000 rpm, resuspended in an equivalent volume of 4% paraformaldehyde in PBS, and incubated for 25 min at room temperature to fix samples. Fixed samples were then centrifuged for 3 min at 15,000 rpm, resuspended in an equivalent volume of PBS, and refrigerated until the time of microscopy. To prepare samples for microscopy, samples were diluted 1:5 in PBS. To prepare agarose pads for imaging, ~400µL of 1% agarose dissolved in PBS was pipetted onto a clean slide and a cover glass was placed on top. Agarose was allowed to harden for ~45min, at which time the cover glass was removed and agar pads were cut into multiple smaller pads. ~1µL of sample was pipetted each agarose pad, allowed to dry, and covered with #1.5 cover glass. Cover glass and slide were bonded with nail polish. Images were acquired with a Nikon TE2000 microscope equipped with a color Nikon Ri2 camera using a 100X Plan Apo 1.4NA objective with DIC optics.

**Protein gels**

Test tubes containing 2mL LB with 100µg/mL AMP (+) or (-) 1mM IPTG were inoculated with scrapings from 25% glycerol, -80°C stocks and incubated 16 h at 37°C with shaking (250rpm). 500µL samples were then centrifuged at 15,000 rpm for 3 min and resuspended in 500µL PBS. To prepare cell lysates, 20µL sample was mixed 1:1 with Laemmli Sample Buffer and incubated at 95°C for 5 min. Samples were centrifuged at 15,000 rpm for 3 min, and 20µL supernatant was loaded into wells of a 12% Mini-PROTEAN® TGX™ precast polyacrylamide gel. Gels were run in a BIO-RAD Mini-PROTEAN® Tetra Cell according to the manufacturer's instructions and stained with Coomassie Brilliant Blue. If bands were to be analyzed by mass spectrometry, bands were excised from the gel.

**Mass spectrometry**

Gel bands were digested using trypsin[12]. Dried gel bands were reconstituted in 0.1% formic acid and analyzed on an Orbitrap Elite™ (ThermoScientific, Bremen, Germany) mass spectrometer coupled to an Easy nLC 1000 UPLC and 50cm long column packed with 1.8µm C18 particles (Dr. Maisch, Germany). MS/MS data was analyzed in Proteome Discoverer (ThermoScientific) using SequestHT[13] and MS-Amanda[14] algorithms against Uniprot *E. coli* database (downloaded 09152015) and appended with common contaminants. Scaffold (version Scaffold_4.4.5, Proteome Software Inc., Portland, OR) was used to validate MS/MS based peptide and protein identifications.

**RNA isolation**

MG1655 bearing $P_{T5}$-*ydcSTUV-patD* or empty vector was inoculated from a 25% glycerol, -80°C stock into a test tube containing 2mL LB with 100µg/mL AMP and grown 4 h at 37°C with shaking (250rpm). Each pregrowth was then diluted to $OD_{600}$~0.01 in two test tubes, each with 2mL LB and 100µg/mL AMP, and incubated at 37°C

with shaking (250 rpm). At t=2 h, 1mM IPTG or the equivalent volume of sterile dI $H_2O$ was added to the appropriate test tubes. Cultures were incubated at 37°C and 250 rpm for 1 h, at which time 350μL of culture was removed and mixed with 700μL RNAprotect® Bacterial Reagent (Qiagen). The manufacturer's protocol was followed to stabilize RNA, and pellets were frozen at -80°C until all RNA samples for all replicates were collected. RNA of all samples was then purified simultaneously. The protocol for enzymatic lysis of bacteria using the RNeasy® Mini Kit (Qiagen) was followed, including the on-column DNase digestion using RNase-free DNase (Qiagen), with the modification of 10μL Proteinase K (Qiagen) added to 100μL TE buffer containing lysozyme, as suggested for samples grown in complex media. RNA samples were stored at -80°C.

**Reverse transcription (cDNA synthesis)**

TaqMan® Reverse Transcription Reagents were used to convert RNA to cDNA. The manufacturer's protocol was followed for a 10μL reaction using random hexamers. As a control to allow for qPCR quantification of contaminating DNA, a second set of reactions were run, excluding the reverse transcriptase enzyme, RNase inhibitor, random hexamers, and dNTP mix.

**Real-time quantitative PCR (qPCR)**

20μL reactions containing 1μL cDNA from RT reaction, 250nM forward and reverse primers (Supplementary Table S2c), and 10μL of SYBR® Green PCR Master Mix were prepared in wells of a MicroAmp® Fast Optical 96-well reaction plate. Real-time PCR was performed using an Applied Biosystems ViAA7 thermocycler run for 40 cycles. Separate reactions were run for *ydcS* and *patD*, as well as the housekeeping gene *rrsA*, which was used for data normalization. Reactions for all targets were run on DNA samples prepared with and without reverse transcriptase ("RT+" and "RT-," respectively) in biological triplicate. Amplification curves provided $C_t$ (threshold cycle) values. Serial dilutions of the $P_{T5}$-*ydcSTUV-patD* plasmid (with primers targeting *ydcS* and *patD*) and isolated MG1655 genomic DNA (with primers targeting *rrsA*) were included on the PCR plate. $C_t$ values from serial dilutions were associated with known concentrations of DNA to provide a standard curve for quantification of targets in samples of interest. Target counts in RT- samples were subtracted from RT+ samples for each target. *ydcS* and *patD* quantities were calculated relative to *rrsA*. For each gene target (*ydcS* and *patD*), the sample with the maximum number of counts was set at 100%, and expression levels in the other strains are presented as relative to this maximum (Supplementary Fig. S7d).

## SUPPLEMENTARY REFERENCES


1 Aldea, M., Hernandez-Chico, C., de la Campa, A. G., Kushner, S. R. & Vicente, M. Identification, cloning, and expression of bolA, an ftsZ-dependent morphogene of Escherichia coli. *J. Bacteriol.* **170**, 5169-5176 (1988).

2 Kohanski, M. A., Dwyer, D. J., Hayete, B., Lawrence, C. A. & Collins, J. J. A common mechanism of cellular death induced by bactericidal antibiotics. *Cell* **130**, 797-810 (2007).

3 Baba, T. *et al.* Construction of Escherichia coli K-12 in-frame, single-gene knockout mutants: the Keio collection. *Mol. Syst. Biol.* **2**, 2006.0008 (2006).

4 Zaslaver, A. *et al.* A comprehensive library of fluorescent transcriptional reporters for Escherichia coli. *Nat. Methods* **3**, 623-628 (2006).

5 Volzing, K. G. & Brynildsen, M.P. Stationary phase persisters to ofloxacin sustain DNA damage and require repair systems only during recovery. *mBio* **6**, e00731-15 (2015).

6 Amato, S. M. & Brynildsen, M. P. Persister heterogeneity arising from a single metabolic stress. *Curr. Biol.* **25**, 2090-2098 (2015).

7 Orman, M. A. & Brynildsen, M. P. Inhibition of stationary phase respiration impairs persister formation in E. coli. *Nat. Commun.* **6**, 7983 (2015).

8 Datsenko, K. A. & Wanner, B. L. One-step inactivation of chromosomal genes in Escherichia coli K-12 using PCR products. *Proc. Natl. Acad. Sci.* **97**, 6640-6645 (2000).

9 Orman, M. A. & Brynildsen, M. P. Dormancy is not necessary or sufficient for bacterial persistence. *Antimicrob. Agents Chemother.* **57**, 3230-3239 (2013).

10 Shen, Y., Siryaporn, A., Lecuyer, S., Gitai, Z. & Stone, H. A. Flow directs surface-attached bacteria to twitch upstream. *Biophys. J.* **103**, 146-151 (2012).

11 Ma, C. *et al.* Energy production genes sucB and ubiF are involved in persister survival and tolerance to multiple antibiotics and stresses in Escherichia coli. *FEMS Microbiol. Lett.* **303**, 33-40 (2010).

12 Shevchenko, A., Tomas, H., Havlis, J., Olsen, J. V. & Mann, M. In-gel digestion for mass spectrometric characterization of proteins and proteomes. *Nat. Protoc.* **1**, 2856-2860 (2007).

13 Eng, J. K., McCormack, A. L. & Yates, J. R. An approach to correlate tandem mass spectral data of peptides with amino acid sequences in a protein database. *J. Am. Soc. Mass Spectr.* **5**, 976-989 (1994).

14 Dorfer, V. *et al.* MS Amanda, a universal identification algorithm optimized for high accuracy tandem mass spectra. *J. Proteome Res.* **13**, 3679-3684 (2014).
